# Regulatory mechanisms, functions, and clinical significance of CircRNAs in triple-negative breast cancer

**DOI:** 10.1186/s13045-021-01052-y

**Published:** 2021-03-06

**Authors:** Lijuan Lyu, Shizhen Zhang, Yujiao Deng, Meng Wang, Xinyue Deng, Si Yang, Ying Wu, Zhijun Dai

**Affiliations:** 1 Department of Breast Surgery, The First Affiliated Hospital, College of Medicine, Zhejiang University, Hangzhou, 310003 China; 2 Department of Oncology, The Second Affiliated Hospital of Xi’an Jiaotong University, Xi’an, China; 3 The Cancer Institute of the Second Affiliated Hospital and Institute of Translational Medicine, Zhejiang University School of Medicine, Hangzhou, China

**Keywords:** CircRNAs, Triple-negative breast cancer, Biogenesis, Biological functions, Regulation mechanisms, Diagnosis, Clinicopathology, Prognosis

## Abstract

Circular RNAs (circRNAs) are a new class of endogenous regulatory RNAs characterized by covalently closed cyclic structure lacking poly-adenylated tails, and are capable of regulating gene expression at transcription or post-transcription levels. Recently, plentiful circRNAs have been discovered in breast cancer and some circRNAs expression profiles are specifically involved in the triple-negative breast cancer (TNBC). TNBC is a type of malignant tumor defined by the lack of estrogen receptor, progesterone receptor, and human epidermal growth factor receptor 2 expression. Considering its clinical characteristics of high invasion, metastasis, poor prognosis, and lack of effective response to conventional chemotherapies or targeted therapies, it could be a promosing option to discover specific circRNAs as new targets for TNBC treatment. Meanwhile, accumulating evidence has demonstrated that circRNAs are dysregulated in TNBC tissues and are correlated with clinicopathological features and prognosis of TNBC patients. Furthermore, looking for circRNAs with high specificity and sensitivity will provide a new opportunity for the early diagnosis, clinical treatment, and prognosis monitoring of TNBC. Herein, we reviewed the biogenesis, regulatory mechanisms, and biological functions of circRNAs in TNBC and summarized the relationship between circRNAs expression and the clinicopathology, diagnosis, and prognosis of patients with TNBC.

**Supplementary Information:**

The online version contains supplementary material available at 10.1186/s13045-021-01052-y.

## Background

Breast cancer (BC) is the most common malignant disease among females and seriously threatens the health of women worldwide [1, 2]. Triple-negative breast cancer (TNBC) is the subtype of BC with the highest recurrence, metastasis, and mortality rate. It is pathologically characterized by the absence of estrogen receptor (ER), progesterone receptor (PR), and human epidermal growth factor receptor 2 (HER2) [3, 4]. TNBC accounts for approximately 15% of all BCs, with typically more aggressive characteristics and lack of effective targeted treatment options [5, 6]. Therefore, early detection and feasible targeted therapy are especially important for TNBC patients. Traditionally, many clinicopathological features, such as tumor size, lymph node status, and histological grade, are associated with patient outcomes and are used to predict patient prognosis [7]. Several newly identified biomarkers, such as tumor-associated macrophages (TAMs), microRNAs (miRNAs), and long non-coding RNA (lncRNAs), also have important prognostic values [8]. In recent years, circular RNAs (circRNAs) have attracted a lot of attention due to their key roles involved in human cancers, including TNBC.

CircRNA was first observed in the 1976 by Sanger et al. in plant-infected viroids by electron microscopy and were considered pathogenic because of their structural similarity to viruses [9]. They were later discovered in eukaryotes and were thought to be a result of splicing errors for several decades after the 1970s [10, 11]. However, more recent studies of circRNAs in drosophila, mouse, and other eukaryotes indicate that these RNA transcripts are evolutionarily conserved and thus are not simple artifacts of faulty splicing [12, 13]. In addition, with the advances in sequencing technology and bioinformatics analyses, the abundance and diversity of circRNAs therefore can been easily identified [14, 15]. High-throughput RNA sequencing (RNA-seq) and microarray are widely used technology to annotate new RNA species and quantify RNA abundance, which have identified the majority of circRNAs in human cells. Besides, increasing bioinformatic algorithms have been developed for identifying circRNAs, such as circRNA_finder, find_circ, CIRCexplorer, CIRI, and MapSplice [16]. The mainly validation methods for circRNA expression are quantitative real-time PCR (qRT-PCR) and Northern blotting, and Northern blotting is a more stringent circRNA validation method than qRT-PCR, given its straightforward procedure with no reverse transcription and amplification steps [17].

Researchers have identified that circRNAs possess significant roles in regulation of multiple factors at transcription or post-transcriptional levels in mammalian cells, and dysregulations of circRNAs can affect genes expression and lead to diseases [18–20], including cancer [21, 22]. Many studies using microarray and RNA-seq revealed that circRNAs can be frequently detected in BC [23, 24]. Especially, recent studies depicted the systematic profiling and characterization of circRNA expression pattern in different subtypes of BC [25, 26], and such subtype-specific set of circRNAs may be used for distinguishing the tumor subtypes, suggesting that circRNAs can be exploited as novel molecular biomarkers. Notably, more and more evidence have indicated that dysregulation of circRNAs participate in carcinogenesis and progression of TNBC, as a result, certain circRNAs could be potentially diagnostic and prognostic biomarkers or therapeutic targets for TNBC [27–29]. Hence, we concentrated on recent findings related to the role of circRNAs in TNBC and summarized their potential clinical implications in TNBC, such as identification of biomarkers for early and differential diagnosis, prognosis, and prediction of response to specific therapies.

## The biogenesis of circRNAs

**Fig. 1:**
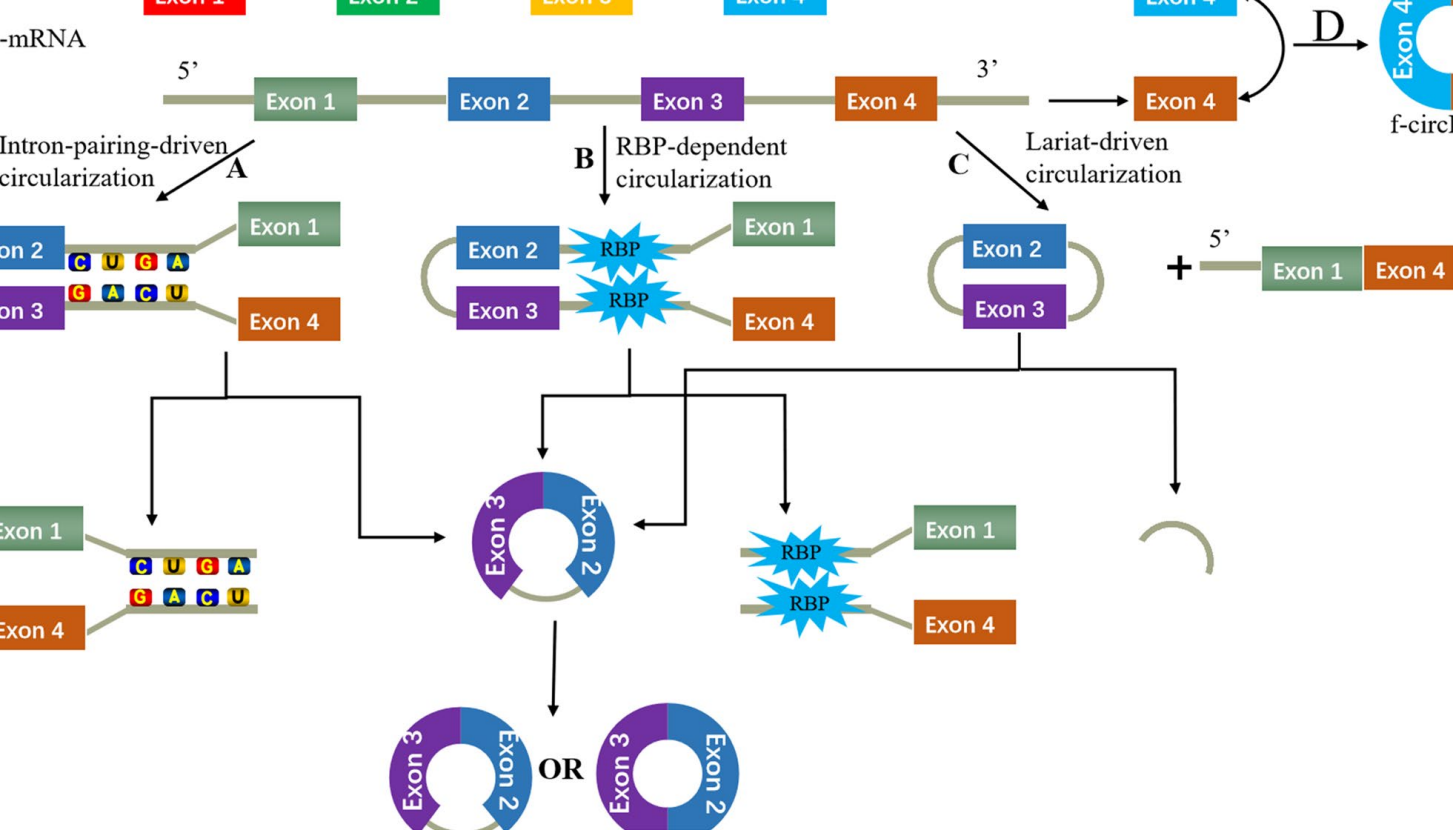
Biogenesis mechanism of EcircRNA and EIciRNA circRNAs. **a** Intron-pairing-driven circularization: the upstream intron pairs with the downstream intron, then the 2′-hydroxyl of the upstream intron reacts with the 5′-phosphate of the downstream intron, followed by the 3′-hydroxyl of the 3′-exon reacting with the 5′-phosphate of the 5′-exon; **b** RBPs-dependent circularization: RNA binding proteins (RBPs) bind the upstream and downstream introns and are attracted to each other, and form a bridge between the introns, then the 2′-hydroxyl of the upstream intron reacts with the 5′-phosphate of the downstream intron, followed by the 3′-hydroxyl of the 3′-exon reacting with the 5′-phosphate of the 5′-exon; **c** Lariat-driven circularization: Folding of a region of pre-RNA can result in exon skipping; furthermore, the splice donor in 3′ end of exon 1 and the splice acceptor in 5′ end of exon 4 are covalently joined together to form a lariat containing exon 2 and exon 3; **d** Fusion-circRNAs contain two exon circRNA fragments flanked by GT-AC splicing signals acting as the splice donor and acceptor of the circular junction while forming an integrated circRNA

**Fig. 2:**
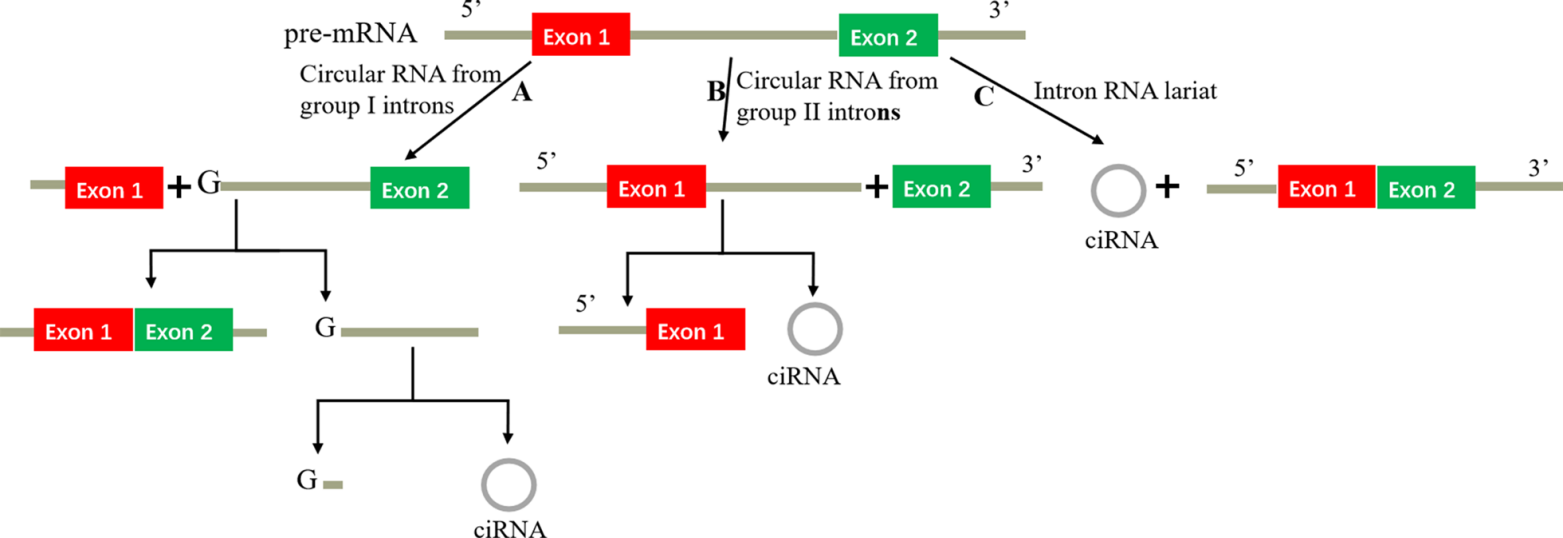
Biogenesis mechanism of intronic circRNA. **a** Circular RNA from group I introns: first, an exogenous guanosine (G) attacks the 5′-terminus of the intron as nucleophile and the 5′-exon is cut off due to the transesterification; Second, the 3′-hydroxyl of the free exon attacks the 5′-terminus of the 3′-exon as nucleophile, producing a linear intron; Third, a 2′-hydroxyl close to the 3′-terminus of the linear intron attacks a phosphodiester bond close to the 5′-terminus, producing an RNA lariat circularized with 2′,5′-phosphodiester and releasing the 5′-terminal sequence; **b** Circular RNA from group II introns: the pre-mRNA releases the 3′-exon, then the 2′-hydroxyl of the 3′-terminus attacks the 5′-terminus of the intron, producing an circular RNA circularized with 2′,5′-phosphodiester; **c** Intron RNA lariat: the pre-mRNA is spliced by a spliceosome, producing an RNA lariat circularized with 2′,5′-phosphodiester

CircRNAs are derived from precursor messenger RNAs (pre-mRNAs), which are transcribed by RNA polymerase II, and characterized by circular shapes resulting from covalently closed continuous loops [30, 31]. With their unique structures, circRNAs are resistant to exonuclease RNase Rand, which makes them more conservative and stable than their linear counterparts [32]. CircRNAs are mainly divided into four types according to their various components and circularization mechanism, including exon circRNAs (EcircRNAs), circular intronic RNAs (ciRNAs), exon–intron circRNAs (EIciRNAs), and intergenic circRNAs or fusion circRNAs (f-circRNAs) [33]. EcircRNAs, consisting of only one exon or multiple quantities of exons and forming through a shearing process called “head-to-tail” or “backsplicing”, make up over 80% of circRNAs and mostly exist in the cytoplasm [34]. EIciRNAs are predominantly located in the nucleus and is circularized in the form of retaining introns between exons [35]. There are currently three models, namely intron-pairing-driven circularization, RNA-binding-protein (RBP)-dependent circularization, and lariat-driven circularization, that have been recognized to elaborate the origination of EcircRNAs and EIciRNAs (Fig. 1) [36]. Notably, a newly discovered type of circRNA termed ciRNAs, are derived from introns and mainly found in the nucleus. There are also three hypothetical models explaining the formation of ciRNAs, including circular RNA from group I introns, circular RNA from group II introns, and intron RNA lariat (Fig. 2) [36, 37]. The f-circRNAs are identifed by applying CIRI (an algorithm for de novo circular RNA identification) and contain two intronic circRNA fragments flanked by GT-AC splicing signals acting as the splice donor and acceptor of the circular junction while forming an integrated circRNA (Fig. 1d) [38].

## Regulatory mechanisms of circRNAs in TNBC

**Fig. 3:**
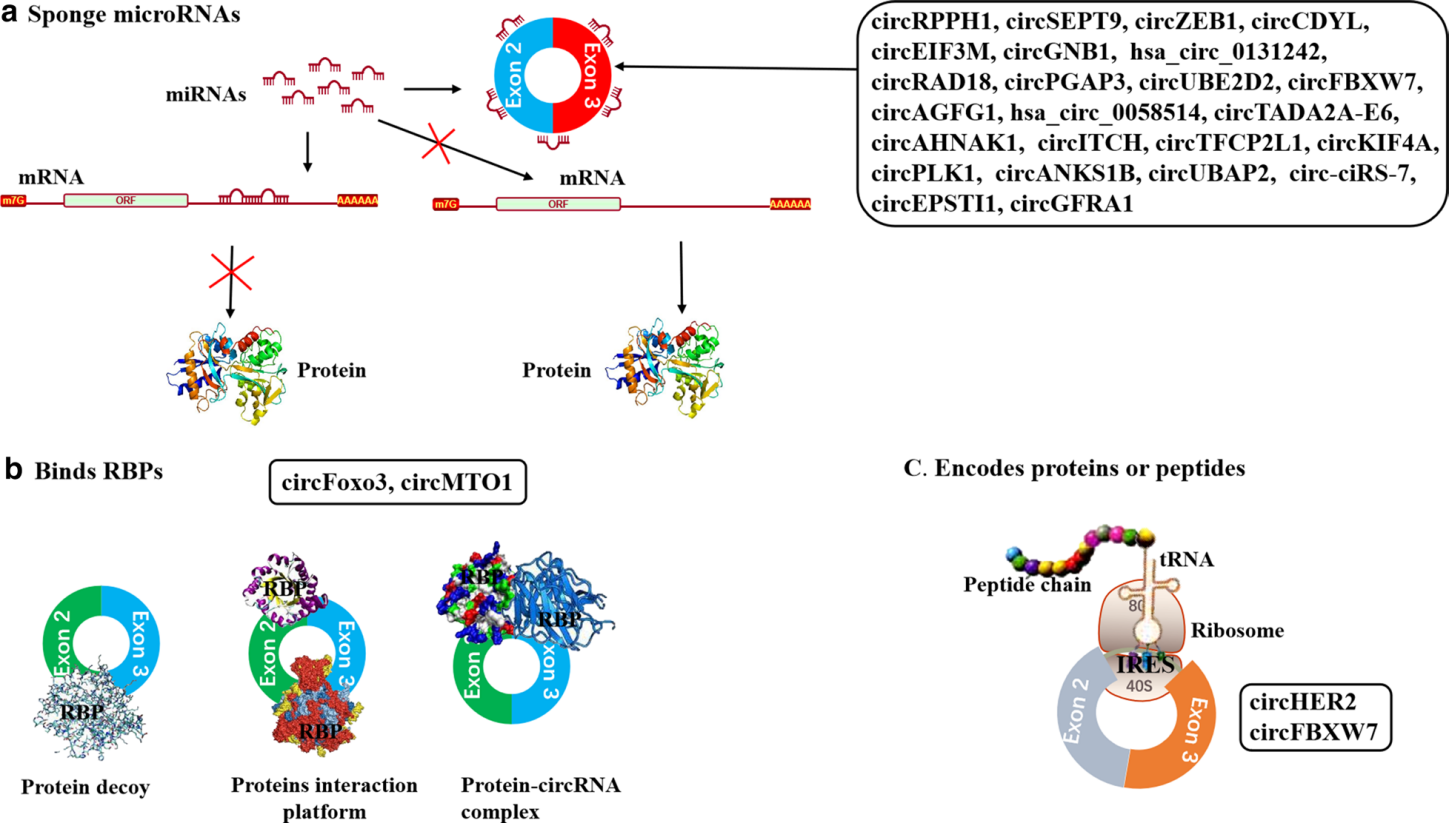
Regulatory mechanisms of circRNAs in TNBC. **a** Serving as miRNA sponge to compete endogenous RNA and sequester miRNAs from binding mRNA targets to influence downstream protein translation; **b** Binding RBPs to block their function (protein decoy), form protein-circRNAs complexes, or be scaffolds for protein–protein interactions; **c** some circRNAs which contains the IRES could be translated into proteins

CircRNAs are a novel class of abundant, stable and ubiquitous noncoding RNAs with diverse regulatory roles in tumor cells, including serving as miRNA sponges, binding to RBPs, modulating genes transcription, competing with linear splicing, translating into protein, and so on [20, 39]. In TNBC, the circRNAs has not yet been found to modulate genes transcription and compete with linear splicing. The regulatory mechanisms of circRNAs involved in TNBC cells are depicted in Fig. 3 and Table 1, which summarized most circRNAs involved in TNBC.

**Table 1:** Summary of dysregulated circRNAs in TNBC

CircRNAs	Location	Technique/cohort	Method	Sample type	Expression in tumor	Roles	Biomarkers	AUC	Survival	Functions	Mechanism	Gene symbol	Pathway	References
circGFRA1	cytoplasm	MDA-MB-231/PTX	qRT-PCR	MDA-MB-231/PTX	Up in MDA-MB-231/PTX	Oncogenic	Prediction of therapy response	NR	NR	Increase the resistance of TNBC cells to PTX	Sponge miR-361-5p	TLR4	NR	[50]
circRPPH1	cytoplasm	GSE101123/20 TNBC versus 20 N	qRT-PCR	TNBC tissues and cells	Up	Oncogenic	NR	NR	NR	Proliferation and metastasis	Sponge miR-556-5p	YAP1	NR	[60]
circSEPT9	cytoplasm	RNA-seq of 4 TNBC versus 4 N/60 TNBC versus 60 N/ 80 TNBC	qRT-PCR, ISH	TNBC tissues and cells	Up	Oncogenic	Diagnosis and prognosis	0.711	OS	Proliferation, metastasis, apoptosis, and autophagy	Sponge miR-637	LIF	NR	[49]
circLARP4	NR	283 BC versus 283 N 65 TNBC versus 65 N	qRT-PCR	TNBC tissues and cells	Down	Antitumor	Prediction of therapy response	NR	NR	Inhibit the resistance of TNBC cells to DOX	NR	NR	NR	[89]
circUSP42	NR	RNA-seq of TNBC versus adjacent tissues/30 TNBC versus 30 N	qRT-PCR	TNBC tissues	Down	Antitumor	Prognosis	NR	OS, DFS	NR	NR	NR	NR	[98]
circABCB10	NR	MDA-MB-231/PTX	qRT-PCR	MDA-MB-231/PTX	Up in MDA-MB-231/PTX	Oncogenic	Prediction of therapy response	NR	NR	Increase the resistance of TNBC cells to PTX	Sponge Let-7a-5p	DUSP7	NR	[95]
circCDYL	NR	BC versus N	qRT-PCR	BC tissues and TNBC cells	Down	Antitumor	NR	NR	NR	Proliferation, migration, and invasion	Sponge miR-190a-3p	TP53INP1	NR	[61]
circZEB1	NR	30 TNBC versus 30 N	RT-qPCR	TNBC tissue, cells	Up	Oncogenic	NR	NR	NR	Proliferation and apoptosis	Sponge miR-448	eEF2k	NR	[58]
circGNB1	cytoplasm	Microarray/222 TNBC	RT-qPCR	TNBC tissue, cells	Up	Oncogenic	Prognosis	NR	OS, DFS	Proliferation and metastasis	Sponge miR-141-5p	IGF1R	NR	[59]
hsa_circ_0131242	NR	Microarray of TNBC versus normal tissues/30 TNBC versus 30 N /120 TNBC	RT-qPCR	TNBC tissue, cells	Up	Oncogenic	Prognosis	NR	OS	Proliferation and metastasis	Sponge miR-2682	NR	NR	[79]
circEIF3M	cytoplasm	RNA-seq of 3 TNBC versus 3 N /20 TNBC versus 20 N	RT-qPCR	TNBC tissue, cells	Up	Oncogenic	NR	NR	NR	Proliferation, metastasis, cell cycle, and apoptosis	Sponge miR-33a	CCND1	NR	[62]
circHER2	cytoplasm	RNA-seq of 5 TNBC versus 5 N /59 TNBC versus 59 N	RT-qPCR	TNBC tissue, cells	Up	Oncogenic	Prognosis and prediction of therapy response	NR	OS	Proliferation and invasion, circHER2 expressing TNBC are sensitive to Pertuzumab	Encode a novel HER2 variant HER2– 103	HER2– 103	Promote EGFR/HER3 interaction and activation	[75]
circPGAP3	cytoplasm	86 TNBC versus 86 N	RT-qPCR	TNBC tissue, cells	Up	Oncogenic	Prognosis	NR	OS,DFS	Proliferation and metastasis	Sponge miR-330-3p	myc	NR	[64]
circUBE2D2	cytoplasm	66 TNBC versus 66 N	RT-qPCR	TNBC tissue, cells	Up	Oncogenic	Prognosis and prediction of therapy response	NR	OS	Proliferation and metastasis, increase the resistance of TNBC cells to DOX	Sponge miR‑512‑3p	CDCA3	NR	[63]
circRAD18	cytoplasm	Microarray of TNBC versus adjacent tissues/31 TNBC versus 31 N/126 TNBC	RT-qPCR	TNBC tissue, cells	Up	Oncogenic	Diagnosis and prognosis	0.752	OS	Proliferation, metastasis, and apoptosis	Sponge miR-208a/3164	IGF1 and FGF2	NR	[78]
hsa_circ_0005320	cytoplasm	RNA-seq of 4 TNBC versus 4 N/20 TNBC versus 20 N	RTFQ-PCR	TNBC tissue, cells	Up	Oncogenic	NR	NR	NR	proliferation, cell cycle, and apoptosis	NR	LIF、P-STAT3	activate LIF-STAT3 pathway	[80]
hsa_circ_069718	NR	Microarray of TNBC versus adjacent tissues/35 TNBC versus 35 N	RT-qPCR	TNBC tissue, cells	Up	Oncogenic	Prognosis	NR	OS	Proliferation and invasion	NR	β-catenin, c-myc, and cyclin D1	Activate Wnt/β-catenin pathway	[81]
circFBXW7	cytoplasm	473 TNBC	RT-qPCR	TNBC tissue, cells	Down	Antitumor	Prognosis	NR	OS, DFS	Proliferation and metastasis	Sponge miR-197-3p and encode the FBXW7-185aa protein	FBXW7	NR	[74]
circCDR1as	NR	MDA-MB-231/5-Fu	RT-qPCR	MDA-MB-231/5-Fu	Up	Oncogenic	Prediction of therapy response	NR	NR	Increase the resistance of TNBC cells to 5-FU	Sponge miR-7	NR	NR	[97]
circAGFG1	cytoplasm	RNA-seq of 4 TNBC versus 4 N/40 TNBC versus 40 N/80 TNBC	RT-qPCR, ISH	TNBC tissue, cells	Up	Oncogenic	Diagnosis and prognosis	0.767	OS	Proliferation, metastasis, cell cycle, and apoptosis	Sponge miR-195-5p	CCNE1	NR	[57]
hsa_circ_0058514	NR	RNA-seq of 4 TNBC versus 4 N/20 TNBC versus 20 N	RTFQ-PCR	TNBC tissue, cells	Up	Oncogenic	NR	NR	NR	Proliferation, metastasis, cell cycle, and apoptosis	NR	CCNE1 and CDK2	NR	[82]
circTADA2A-E6	cytoplasm	Microarray of 8 specimens (4 TNBC and 4 luminal A) versus 3 normal tissues/178 BC versus 16 N /115 TNBC	RT-qPCR	TNBC tissue, cells	Down	Antitumor	Diagnosis and prognosis	0.8554	OS, DFS	Proliferation and metastasis	Sponge miR-203a-3p	SOCS3	NR	[48]
circAHNAK1	cytoplasm	Microarrays/20 TNBC versus 20 N/136 TNBC	RT-qPCR	TNBC tissue, cells	Down	Antitumor	Diagnosis and prognosis	0.72	OS, DFS	Proliferation and metastasis	Sponge miR-421	RASA1	NR	[56]
circITCH	NR	275 BC versus 68 N/91 TNBC	RT-qPCR	TNBC tissue, cells	Down	Antitumor	Prognosis	NR	OS	Proliferation and metastasis	Sponge miR-214/ miR-17	ITCH	Inactivate the Wnt/β-catenin pathway	[52]
circTFCP2L1	cytoplasm	Microarray of 3 TNBC versus 3 N/32 TNBC versus 32 N	RT-qPCR	TNBC tissue, cells	Up	Oncogenic	Prognosis	NR	DFS	Proliferation and migration	Sponge miR-7	PAK1	NR	[55]
circKIF4A	cytoplasm	Microarray/240 TNBC	RT-qPCR	TNBC tissue, cells	Up	Oncogenic	Prognosis	NR	OS, DFS	Proliferation and metastasis	Sponge miR-375	KIF4A	NR	[51]
circAMOTL1	NR	MDA-MB-231/PTX	qRT-PCR	MDA-MB-231/PTX	Up in MDA-MB-231/PTX	Oncogenic	Prediction of therapy response	NR	NR	Increase the resistance of TNBC cells to PTX	NR	BCL2, BAX and BAK	AKT pathway,	[96]
circKDM4C	cytoplasm	MDA-MB-231/DOX	qRT-PCR	MDA-MB-231/DOX	Down MDA-MB-231/DOX	Antitumor	Prediction of therapy response	NR	NR	Decreased the resistance of TNBC cells to doxorubicin	Sponge miR-548p	PBLD	NR	[90]
circPLK1	cytoplasm	Microarray of four cell lines (MDA-MB-231, MDA-MB-468, BT549 versus MCF-10A)/57 TNBC versus 57 N/240 TNBC	RT-qPCR	TNBC tissue, cells	Up	Oncogenic	Prognosis	NR	OS, DFS	Proliferation and metastasis	Sponge miR-296-5p	PLK1	NR	[54]
circANKS1B	cytoplasm	RNA-seq of 3 TNBC versus 3 N/20 TNBC versus 20 N/ 165 BC versus 40 N	RT-qPCR	BC tissue, cells	Up	Oncogenic	Prognosis	NR	OS	Invasion and metastasis	Sponge miR-148a/ 152-3p	USF1	Activate of TGF-β1 signaling pathway	[47]
circUBAP2	cytoplasm	78 TNBC versus 78 N	RT-qPCR	TNBC tissue, cells	Up	Oncogenic	Prognosis	NR	OS	Proliferation, metastasis, and apoptosis	Sponge miR-661	MTA1	NR	[53]
circ-ciRS-7	cytoplasm	32 TNBC versus 32 N	RT-qPCR	TNBC tissue, cells	Up	Oncogenic	NR	NR	NR	Invasion and metastasis	Sponge miR-1299	MMPs	NR	[45]
circMTO1	nucleus	MDA-MB-231/monastrol	qRT-PCR	MDA-MB-231/monastrol	Down MDA-MB-231/ monastrol	Antitumor	Prediction of therapy response	NR	NR	Reverses the resistance of TNBC cells to monastrol	Binding to TRAF4	Eg5	NR	[67]
circEPSTI1	NR	Microarray of 3 TNBC versus 3 N/37 TNBC versus 30 N/240 TNBC	RT-qPCR, ISH	TNBC tissue, cells	Up	Oncogenic	Prognosis	NR	OS, DFS	Proliferation and apoptosis	SPONGE miR-4753/6809	BCL11A	NR	[46]
circGFRA1	cytoplasm	Microarray of TNBC cells and MCF-10A /51 TNBC versus 51 N/222 TNBC	RT-qPCR	TNBC tissue, cells	Up	Oncogenic	Prognosis	NR	OS, DFS	Proliferation and apoptosis	Sponge miR-34a	GFRA1	NR	[29]
hsa_circ_0006528	NR	MDA-MB-231/DOX	qRT-PCR	MDA-MB-231/DOX	Up in MDA-MB-231/DOX	Oncogenic	Prediction of therapy response	NR	NR	Increase the resistance of TNBC cells to PTX	Sponge miR-7–5p	Raf1	NR	[87]

*TNBC* triple-negative breast cancer, *N* normal, *DOX* doxorubicin, *PTX* paclitaxel, *5-FU* 5-fluorouracil, *MDA-MB-231/PTX* PTX-resistant MDA-MB-231, *MDA-MB-231/DOX* DOX-resistant MDA-MB-231, *MDA-MB-231/ monastrol* monastrol-resistant MDA-MB-23, *5-Fu-resistant MDA-MB-231* MDA-MB-231/5-FU, *OS* overall survival, *DFS* disease-free survival, *AUC* area under the curve, *NR* not report, *ISH* in situ hybridization, *qRT-PCR* quantitative real-time PC

### CircRNAs serve as miRNA sponges

MiRNAs negatively regulate the gene expression of messenger RNAs (mRNAs) through direct base pairing to target sites in mRNA 3′ untranslated regions, eventually leading to decreased mRNA stability and translation suppression [40]. The competing endogenous RNA (ceRNA) hypothesis showed that other RNAs with miRNA target sites can compete with mRNAs for miRNA binding [41]. Indeed, most circRNAs, containing a large number of different types of miRNA response elements, are located in the cytoplasm with huge miRNA-binding capacity and have been found to interact with miRNA and serve as miRNA sponges to remove the inhibitory effect of miRNA on its target genes in cancer [42–44].

Most of the circRNAs currently reported in TNBC serve as miRNA sponges (Fig. 3a). For instance, *ciRS-7*, an earlier discovered circRNA in TNBC, contains 20 *miR-1299*–binding sites and functions as a ceRNA of *miR-1299* to enhance the expression of the *matrix metalloproteinase* family members, thereby contributing to the high migration and invasion properties of TNBC cells [45]. *CircEPSTI1* promotes TNBC proliferation and apoptosis by upregulating *BCL11A* expression via binding to *miR-4753* and *miR-6809* [46]. Zeng et al. [47] reported that *circANKS1B* abundantly sponged *miR-148a-3p* and *miR-152-3p* to increase the expression of transcription factor *USF1*. Moreover, the splicing factor *ESRP1*, regulated by *USF1*, can promote *circANKS1B* biogenesis in TNBC. *CircTADA2A-E6* preferentially acts as an *miR-203a-3p* sponge to restore the expression of miRNA target gene *SOCS3*, resulting in a less aggressive oncogenic phenotype [48]. Zheng et al. demonstrated that *circSEPT9* could regulate the expression of *LIF* via sponging *miR-637* and activating the LIF/Stat3 signaling pathway involved in the progression of TNBC. More importantly, they discovered that E2F1 and EIF4A3 enhance the expression of *circSEPT9* by binding to the *SEPT9* promoter and pre-mRNA [49]. *CircGFRA1* was found not only could upregulate *TLR4* via sponging for *miR-361-5p*, thus affecting the sensitivity of TNBC cells to paclitaxel (PTX) [50], but also upregulate its parent gene *GFRA1* expression through sponging *miR-34a*, thus promoting proliferation and inhibiting apoptosis of TNBC cells [29]. Likewise, *circKIF4A* and *circITCH* were proved to upregulate its parental genes via acting as sponges for miRNAs thereby mediating TNBC progress [51, 52]. Additionally, more miRNA-sponge functions of circRNAs have been and are being validated in TNBC, including *circUBAP2/miR-661/MTA1* [53], *circPLK1/miR-296-5p/PLK1* [54], *circTFCP2L1/miR-7/PAK1* [55], *circAHNAK1/miR-421/RASA1* [56], *circAGFG1/miR-195-5p/CCNE1* [57], *circZEB1/miR-448/eEF2K* [58], *circGNB1/miR-141-5p/IGF1R* [59], *circRPPH1/miR-556-5p/YAP1* [60], *circCDYL/miR-190a-3p/TP53INP1* [61], *circEIF3M/miR-33a/cyclinD1* [62], *circUBE2D2/miR-512-3p/CDCA3* [63], and *circPGAP3/miR-330-3p/Myc* [64].

### CircRNAs interact with RBPs

CircRNAs could specifically bind to proteins directly or through RNA as well as sequester proteins to block the protein effects by working as competing elements (Fig. 3b) [65]. One classic example of circRNA to interact with proteins is *circFoxo3.* It’s expression significantly promoted TNBC cell apoptosis with upregulation of Foxo3, but downregulation of p53. Mechanically, *circFoxo3* prefered to bind MDM2 and p53, instead of Foxo3 in MDA-MB-231 cells. As such, *circFoxo3* overexpression promoted MDM2-induced p53 ubiquitination and subsequent degradation, but competitively prevented MDM2-mediated Foxo3 ubiquitination and degradation, eventually leading to cell apoptosis due to upregulation of the Foxo3 downstream target *PUMA* [66]. Moreover, circRNAs are able to bind and sequester proteins. For example, *circMTO1* interacted with TRAF4 by serving as a ceRNA to repress TRAF4 from binding to the *Eg5* gene, leading to sequester TRAF4 from activating *Eg5* translation, thus mediating TNBC cell resistance to monastrol [67].

### CircRNAs encode proteins

CircRNAs were previously regarded as a distinct class of endogenous non-coding RNAs that could not translate proteins due to lack of 5–3 polarity, a polyadenylated tail, and an internal ribosome entry site (IRES). However, recent studies indicated that some cytoplasmic circRNAs can be effectively translated into detectable peptides [68, 69]. IRES- and N[6]-methyladenosines-mediated cap-independent translation initiation have been suggested to be potential mechanism for circRNA translation [70, 71]. To date, several circRNAs have been uncovered to have the potential to be translated into proteins, for instance, *circZNF609*, *circPABPN1* [72, 73]. In TNBC (Fig. 3c), *circFBXW7* not only can serve as a sponge of *miR-197-3p* to upregulate its parent gene *FBXW7*, but also encode the FBXW7-185aa protein to increase the abundance of FBXW7, thereby promoting c-Myc ubiquitination and degradation, eventually suppressing TNBC cells growth and metastasis [74]. More recently, Li et al. confirmed that a newly identified *HER2* transcriptional variant, *circHER2*, had an open reading frame driven by an IRES and could generate a 103 amino acid protein HER2–103. HER2–103 could promote homo/hetero dimerization of epidermal growth factor receptor (EGFR)/HER3 and sustain AKT phosphorylation and downstream malignant phenotype [75]. Whith the increasing evidence prove that circRNAs could translate proteins directly [72, 73, 76, 77], the notion of circRNAs are non-coding RNAs is becoming doubtful.

## Role of circRNAs on the biological functions of TNBC

**Fig. 4:**
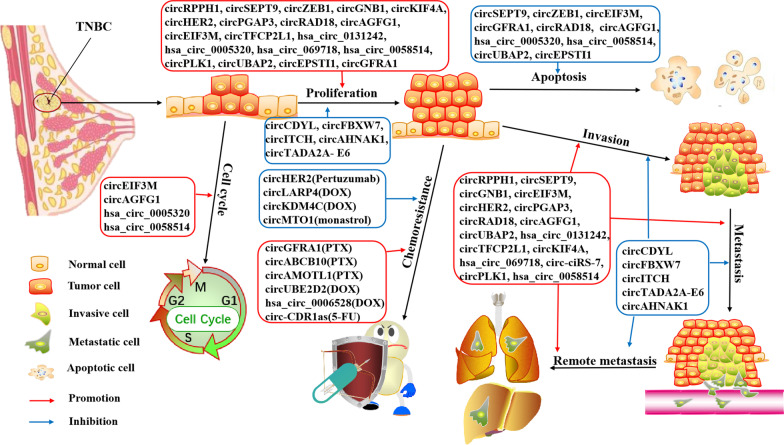
Summary of roles of circRNAs on TNBC cells biological processes. circRNAs play multifaceted roles in TNBC initiation and development, which can control cell proliferation, invasion, metastasis, apoptosis, cell cycle, and chemoresistance by orchestrating their downstream targets. Obviously, certain circRNAs tends to affect TNBC progression by regulating multiple biological processes

CircRNAs play an important role in the regulation of cell proliferation, invasion, metastasis, apoptosis, autophagy, cell cycle, vascularization, and chemoresistance of TNBC by regulating the expression of target genes involved in cancer-related signaling pathways directly or indirectly. The biological roles of circRNAs involved in TNBC cells are depicted and summarized in Fig. 4.

### CircRNAs modulate TNBC proliferation and tumor growth

Majority circRNAs identified in TNBC are characterized by oncogenic features. Specifically, *circRPPH1*, *circSEPT9*, *circGNB1*, *circPGAP3*, *circUBE2D2*, *circRAD18*, *circAGFG1*, *circKIF4A*, *circPLK1*, *circUBAP2*, *circEPSTI1*, and *circGFRA1* were upregulated in both TNBC cells and tissues, and high expression of these circRNAs was able to promote tumor cell proliferation both in vitro and in vivo, and was associated with larger tumor sizes and shorter survival times for TNBC patients [29, 46, 49, 51, 53, 54, 57, 59, 60, 63, 64, 78]. Similarly, *circZEB1*, *circEIF3M*, *circHER2*, *hsa_circ_0131242*, *hsa_circ_0005320*, *hsa_circ_069718*, *hsa_circ_0058514*, and *circTFCP2L1* were overexpressed in TNBC cells and tissues, and they appeared to promote cell proliferation and tumor growth of TNBC [55, 58, 62, 75, 79–82]. On the contrary, a few circRNAs were identified to have tumor-suppressive effects in TNBC. For example, *circFBXW7, circTADA2A-E6, circITCH,* and *circAHNAK1* were found to be downregulated in TNBC cells and tissues. Their expression was negatively correlated with the tumor sizes and DFS or OS of TNBC patients, and ectopic overexpression of these circRNAs obviously inhibited cell proliferation and tumor growth [48, 52, 56, 74]. Lisewise, *circCDYL* was down-regulated in TNBC cells and inhibited proliferation of TNBC cells [61].

### CircRNAs affect invasion and metastasis of TNBC

Certain circRNAs also play pivotal roles in promoting the invasion and metastasis of TNBC. High expression of *circSEPT9*, *circGNB1*, *circAGFG1*, *circPGAP3*, *circKIF4A*, *circPLK1*, *circANKS1B*, *circUBAP2*, and *ciRS-7* significantly contributed to the invasion and metastasis of TNBC cells both in vitro and in vivo, and were correlated with advanced TNM stage and poor prognosis of TNBC patients [45, 47, 49, 51, 53, 54, 57, 59, 64]. Likewise, *circRPPH1*, *hsa_circ_0131242*, *circEIF3M*, *circHER2*, *circUBE2D2*, *circRAD18*, *hsa_circ_069718*, *hsa_circ_0058514*, and *circTFCP2L1* also significantly promoted the migration and invasion capability of TNBC cells in vitro [55, 60, 62, 63, 75, 78, 79, 81, 82]. Conversely, the expression of *circFBXW7, circAHNAK1, circTADA2A-E6,* and *circITCH* appeared to be downregulated in TNBCs and was associated with advanced TNM stage and poor survival for TNBC patients [48, 52, 56, 74]. Ectopic overexpression of *circFBXW7*, *circAHNAK1*, and *circITCH* markedly inhibited the migration of TNBC cells in vitro and obviously reduced the size and number of lung metastasis nodules in xenograft models of TNBC [52, 56, 74]. Epithelial-to-mesenchymal transition (EMT) is a process characterized by the loss of the polarity and adhesion capacity of epithelial cells, but an increase in the mesenchymal traits [83],whcih is pivotal for TNBC cells to metastasize [84]. Notably, *circRPPH1*, *hsa_circ_069718*, *circKIF4A*, and *circPLK1* could increase the expression of mesenchymal marker vimentin and decrease the expression of epithelial marker E-cadherin, thus contributing to EMT and metastasis [51, 54, 60, 81]. Wnt/β-catenin pathway is a key signaling cascade tightly associated with cancer progression. Activation of the Wnt/β-catenin pathway could promote tumor invasion by the upregulation of factors regulating the EMT processes [85]. We found that *hsa_circ_069718* and *circITCH* have opposite roles in regulating the Wnt/β-catenin pathway. *Hsa_circ_069718* activated the Wnt/β-catenin pathway by upregulating *β-catenin*, *c-Myc*, and *cyclin D1* and thus promoted the invasion and metastasis of TNBC cells, while *circITCH* played the opposite role [52, 81]. Besides, *circANKS1B* was proved to promote EMT via increasing the expression of transcription factor *USF1*, which could transcriptionally upregulate *TGF-β1* expression, resulting in activating *TGF-β1/Smad* signaling [47]. On the contrary, *circTADA2A-E6* exerted a negative effect on the regulation of metastasis by suppressing the EMT process [48]. Above all, these circRNAs might act as potential predictors and therapeutic targets for metastatic TNBC.

### CircRNAs regulate apoptosis of TNBC cells

*CircSEPT9*, *circZEB1*, *circEIF3M*, *hsa_circ_0005320*, *circRAD1*8, *circGFRA1*, *hsa_circ_0058514*, *circAGFG1*, *circUBAP2*, and *circEPSTI1* have been proven to be upregulated in TNBC cells and tissues, and associated with decreased cell apoptosis rates of TNBC cells [29, 46, 49, 53, 57, 58, 62, 78, 80, 82]. Silencing of *circSEPT9*, *hsa_circ_0005320,* or *circAGFG1* leads to typical apoptotic morphological characteristics in TNBC cells, such as nuclear shrinkage as well as apoptotic body and nuclear fragmentation [49, 57, 80]. Mechanically, knockdown of *circSEPT9, circZEB1,* or *circAGFG1* could increase the protein levels of the apoptotic markers (*cleaved caspase 3* and *Bax*) while decrease the anti-apoptotic marker (*Bcl-2*) levels in TNBC cells [49, 57, 58]. Taken together, these circRNAs may mediate the progress of TNBC by suppressing tumor cell apoptosis.

### Cell cycle/autophagy/angiogenesis-associated circRNA in TNBC

It is well known that CCNE1 works by forming a complex with CDK2, and the CCNE1-CDK2 complex is able to pushing cell cycle from G1 to S phase, thereby regulating tumor progression [86]. *Hsa_circ_0058514* and *circAGFG1* were proved significantly up-regulated in TNBC cells and tissues and could promote the *CCNE1* and *CDK2* expression via acting as miRNAs sponge, the knockdown of *hsa_circ_0058514* and *circAGFG1* resulted in G1/S phase cell cycle arrest [57, 82]. Similarly, *circEIF3M* acts as a ceRNA to upregulate CCND1, which mainly coordinates with cyclin-dependent kinase 4 (CDK4) to regulate cell cycle progression, downregulation of circEIF3M led to G1 arrest [62]. Besides, silencing *hsa_circ_0005320* led to higher percentages of TNBC cells being arrested in the G1 phase, with lower percentages of cells in the S phase, suggesting *hsa_circ_0005320* also exerts functions in the regulation of the cell cycle of TNBC cells [80]. Meanwhile, Yang et al. also discovered that *circAGFG1* significantly promoted tumor angiogenesis, suggesting *circAGFG1* also play important roles in the regulation of tumor angiogenesis [57]. *CircRPPH*1 was proved to facilitates angiogenesis in TNBC as well [60]. As for autophagy, the knockdown *circSEPT9* in TNBC cells could increase the conversion of the autophagy marker *LC3* from *LC3-I* to *LC3-II* and upregulate the autophagy-related proteins *ATG5* and *ATG7*, thereby inducing *LC3II* punctuation and accumulation of autophagosomes [49].

### CircRNAs regulate TNBC resistance to therapeutic drugs

Chemotherapy is a critical strategy for TNBC treatment, which is usually administered as postoperative therapy or preoperative neoadjuvant therapy. The most commonly used chemotherapeutics, including anthracyclines (doxorubicin[DOX] and epirubicin), taxanes (PTX and docetaxel), 5-fluorouracil (5-FU), and cyclophosphamide, have achieved substantial advantages for TNBC patients, but do not work out for every patient due to drug resistance [4]. CircRNAs have been reported to play vital roles in drug resistance, either by promoting or reversing TNBC chemoresistance [87].

DOX-based chemotherapy is the most frequently used treatment for TNBC [88]. *Hsa_circ_0006528*, *circKDM4C*, *circUBE2D2*, and *circLARP4* were found to be associated with DOX resistance in TNBC [63, 87, 89, 90]. Specifically, *hsa_circ_0006528* was significantly upregulated in DOX-resistant MDA-MB-231 (MDA-MB-231/DOX) cells [87], mechanically by participating in the *circ_0006528/miR-7-5p/Raf1* axis that confers chemotherapeutic resistance in TNBC [91]. Likewise, *circUBE2D2* decreased DOX-induced TNBC cells apoptosis by upregulating CDCA3, which is a trigger of mitotic entry to withstand the DOX-induced apoptosis, indicating that *circUBE2D2* promotes DOX resistance of TNBC cell [63]. Instead, c*ircLARP4* was downregulated in TNBC cell lines, and ectopic overexpression of *circLARP4* can increase the sensitivity of MDA-MB-231 cell lines to DOX [89]. In addition, *circKDM4C* experession was significantly decreased in MDA-MB-231/DOX cells and could attenuate DOX resistance by upregulating *PBLD* [90], which is a tumor suppesssor that could inhibit tumor growth [92].

Chemoresistance against PTX is one of the major issues related to treatment failure in TNBC patients. However, the mechanism by which TNBC cells become resistant to PTX remains unclear. Recently, three circRNAs, *CircGFRA1, circABCB10* and *circAMOTL1*, were identified as important factors that may be responsible for the adverse resistance to PTX in TNBC cells. Specifically, *CircGFRA1* and *circABCB10* were upregulated in the PTX-resistant MDA-MB-231 (MDA-MB-231/PTX) cells. *CircGFRA1* knockdown can inhibit the resistance of TNBC cells to PTX by reducing the expression of *TLR4*, which has been found to be activated by paclitaxel to improve tumor cell survival and blocking TLR4 could significantly improve response to paclitaxel therapy in BC [50, 93]. *CircABCB10* contributed to PTX resistance of TNBC cells through up-regulating of *DUSP7*, which exerts its function by dephosphorylating MAPK [94, 95]. Besides, *circAMOTL1* promoted the chemoresistance against PTX in TNBC cells via posttranscriptional regulation of AKT and therefore led to increase the anti-apoptotic gene *BCL2* expression and inhibit the pro-apoptotic gene *BAX* and *BAK* expression [96]].

Moreover, *circ-CDR1as* was found to be associated with 5-FU-resistant in MDA-MB-231 cells by inhibiting miR-7 to upregulate *CCNE1* [97]. Besides, *circMTO1*, which is usually downregulated in monastrol-resistant MDA-MB-231 cells, can promote monastrol-induced cytotoxicity by targeting Eg5 and sequestering *TRAF4* from binding to the *Eg5* gene [67]. Interestingly, *circHER2*, which encodes a novel protein HER2–103, was proved to be expressed in some TNBC samples, and HER2–103-positive TNBC cells were sensitive to Pertuzumab due to HER2–103 shared the same amino acid sequences as the HER2 CR1 domian [75].

More and more circRNAs are being identified to be associated with chemoresistance, however, our understanting of the mechanistic role of circRNAs contributing to chemotherapeutic resistance is limited due to lack of deep mechanistic investigations and in vivo studies. Whether circRNAs could be a potential target for overcoming TNBC chemoresistance requires further exploration.

## Clinical significance of circRNAs in TNBC

CircRNAs have also been proven to possess potential values for diagnosis and prognosis of TNBC. As a result, circRNAs have received considerable interest for their potential as prognostic markers or therapeutic targets.

### CircRNAs acts as diagnostic biomarkers for TNBC

There are six circRNAs have been currently identified to have diagnostic values in TNBC. Among them, three circRNAs were upregulated and the other three were downregulated in TNBC (Table 2). *CircAHNAK1* was the first identified circRNA that significantly downregulated in TNBC tissues, therefore it can be used as a diagnostic indicator for distinguishing TNBC from normal breast tissue [56]. Xu et al. [48] identified two differentially expressed circTADA2As, *circTADA2A-E6* and *circTADA2A-E5/E6*, that were spliced from exon 6 or exons 5 and 6 of the same *TADA2A* gene respectively, in TNBC, and found that these two circRNAs were significantly downregulated in TNBC and exhibited excellent diagnostic values. Besides, *circAGFG1*, *circRAD18*, and *circSEPT9* were found to be upregulated in TNBC and also exhibited excellent ability in discriminating between TNBC patients and normal individuals [49, 57, 78].

**Table 2:** Summary of diagnosis values of circRNAs in TNBC

CircRNAs	TNBC samples	Normal samples	Expression in TNBC	Method	Sample type	AUC	Sensitivity	Specificity	References
circSEPT9	60	60	Up	RT-qPCR	Tissue	0.711	0.633	0.75	[49]
circRAD18	31	31	Up	RT-qPCR	Tissue	0.752	NR	NR	[78]
circAGFG1	40	40	Up	RT-qPCR	Tissue	0.767	NR	NR	[57]
circTADA2A-E6	115	16	Down	RT-qPCR	Tissue	0.8554	NR	NR	[48]
circTADA2A-E5/E6	115	16	Down	RT-qPCR	Tissue	0.9366	NR	NR	[48]
circAHNAK1	20	20	Down	RT-qPCR	Tissue	0.72	NR	NR	[56]

*TNBC* triple-negative breast cancer, *NR* not report, *qRT-PCR* quantitative real-time PCR, *AUC* area under the curve

### Association of circRNAs with clinicopathological features of TNBC

**Fig. 5:**
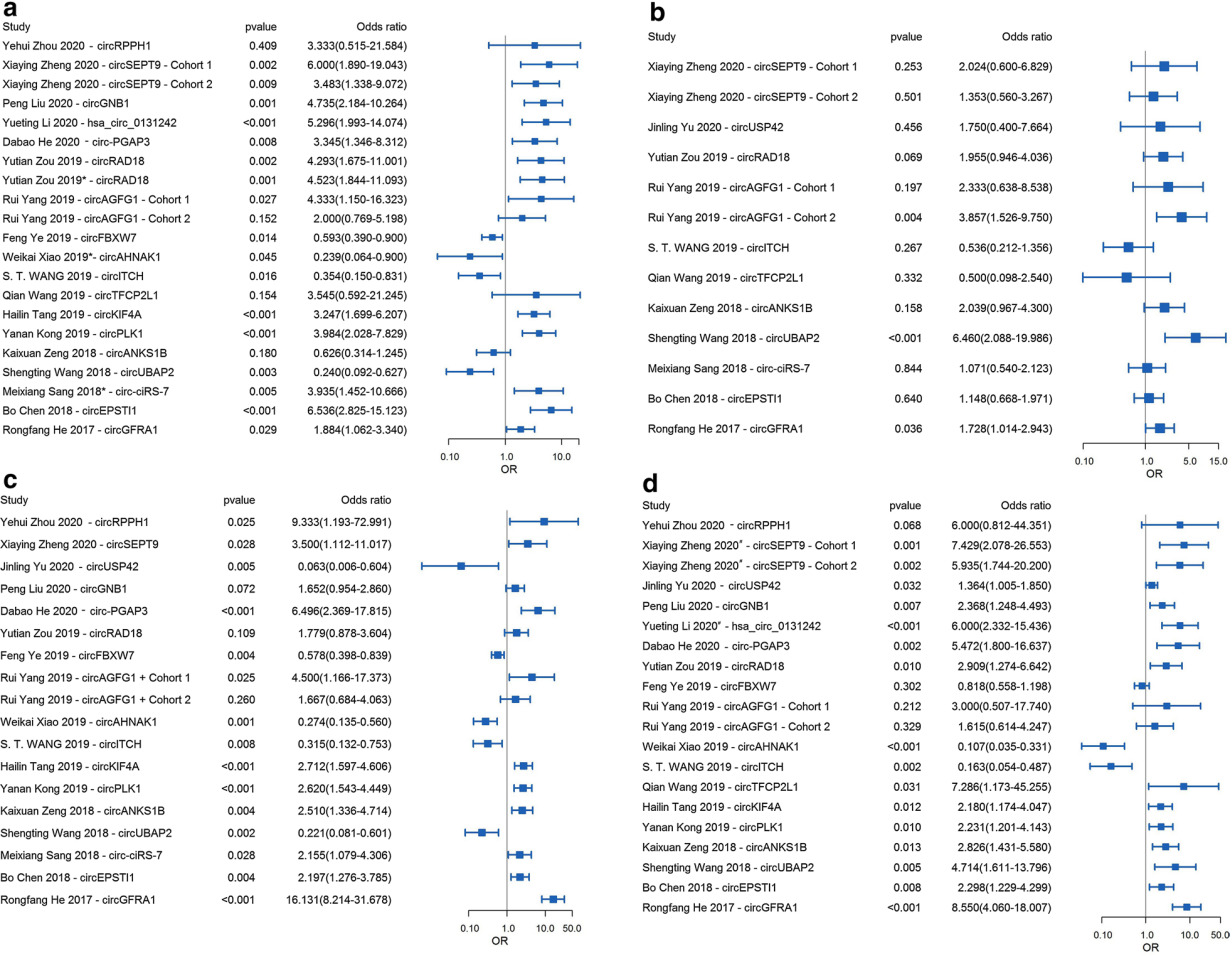
Forest plots of the associations between the expression of circRNAs and **a** tumor sizes (> 2 vs ≤ 2), **b** histological grade (III vs I + II), **c** lymph node metastasis (positive vs negative), **d** TNM stage (III + IV vs I + II) of TNBC. Each square indicates a study. * indicates > 5 versus ≤ 5, # indicates III + II versus I; *OR* odds ratio, *CI* confidence interval

Based on the current reported evidences, we summarized the relationship between circRNAs expression and the clinicopathological factors of TNBC in Additional file 1: Tables S1 and S2. Accordingly, there was no significant correlation between any circRNAs expression and age or menopause status of TNBC patients (Additional file 2: Figure 1). While many circRNAs expression was significantly associated with other clinical parameters of TNBC, including tumor size, lymph node metastasis, histological grade, and TNM stage (Table 3). More importantly, the expression levels of *circSEPT9*, *circGNB1*, *hsa_circ_0131242*, *circPGAP3*, *circRAD18*, *circAGFG1*, *circKIF4A*, *circPLK1*, *circUBAP2*, *circ-ciRS-7*, *circEPSTI1*, and *circGFRA1* were positively correlated with the tumor size [29, 45, 46, 49, 51, 53, 54, 57, 59, 60, 64, 78, 79], whereas *circFBXW7*, *circAHNAK1*, and *circITCH* presented negative associations (Fig. 5a) [52, 56, 74]. *CircAGFG1* and *circGFRA1* were positively related to the histological grade of TNBC (Fig. 5b) [29, 57]. High expressions of *circRPPH1*, *circSEPT9*, *circAGFG1*, *circPGAP3*, *circUBE2D2*, *circKIF4A*, *circPLK1*, *circANKS1B*, *circUBAP2*, *circ-ciRS-7*, *circEPSTI1*, and *circGFRA1* were associated with positive lymph node metastasis [29, 45–47, 51, 53, 54, 57, 60, 63, 64], while low expressions of *circUSP42*, *circFBXW7*, *circTADA2A-E6*, *circAHNAK1*, and *circITCH* were associated with positive lymph node metastasis in TNBC (Fig. 5c) [48, 52, 56, 74]. Besides, increased expression of *circSEPT9*, *circGNB1*, *hsa_circ_0131242*, *circPGAP3*, *circUBE2D2*, *circRAD18*, *hsa_circ_069718*, *circKIF4A*, *circPLK1*, *circANKS1B*, *circUBAP2*, and *circEPSTI1* in TNBC suggested advanced TNM stage [46, 47, 49, 51, 53, 54, 59, 63, 64, 78, 79, 81], whereas decreased expression of *circUSP42*, *circTADA2A-E6, circAHNAK1,* and *circITCH* in TNBC indicated advanced TNM stage (Fig. 5d) [48, 52, 56].

**Table 3:** Summary of circRNAs related to clinicopathological features of TNBC

Clinicopathological factors	Correlation of circRNAs expression with clinicopathological factors in TNBC	
	Positive	Negative
Tumor size	circSEPT9, circGNB1, hsa_circ_0131242,circPGAP3, circRAD18, circAGFG1, circKIF4A, circPLK1, circUBAP2, circ-ciRS-7, circEPSTI1, circGFRA1	circFBXW7, circAHNAK1, circITCH
Histological grade	circAGFG1, circGFRA1	NR
LN metastasis	circRPPH1, circSEPT9, circPGAP3, circUBE2D2, circAGFG1, circKIF4A, circPLK1, circANKS1B, circUBAP2, circ-ciRS-7, circEPSTI1, circGFRA1	circUSP42, circFBXW7,circTADA2A-E6,circAHNAK1,circITCH
TNM stage	circSEPT9, circGNB1, hsa_circ_0131242, circPGAP3, circUBE2D2, circRAD18, hsa_circ_069718, circKIF4A, circPLK1,circANKS1B, circUBAP2, circEPSTI1	circUSP42, circTADA2A-E6,circAHNAK1,circITCH

*TNBC* triple-negative breast cancer, *NR* not report, *LN* lymph node

### CircRNAs act as prognostic biomarkers for BC

**Fig. 6:**
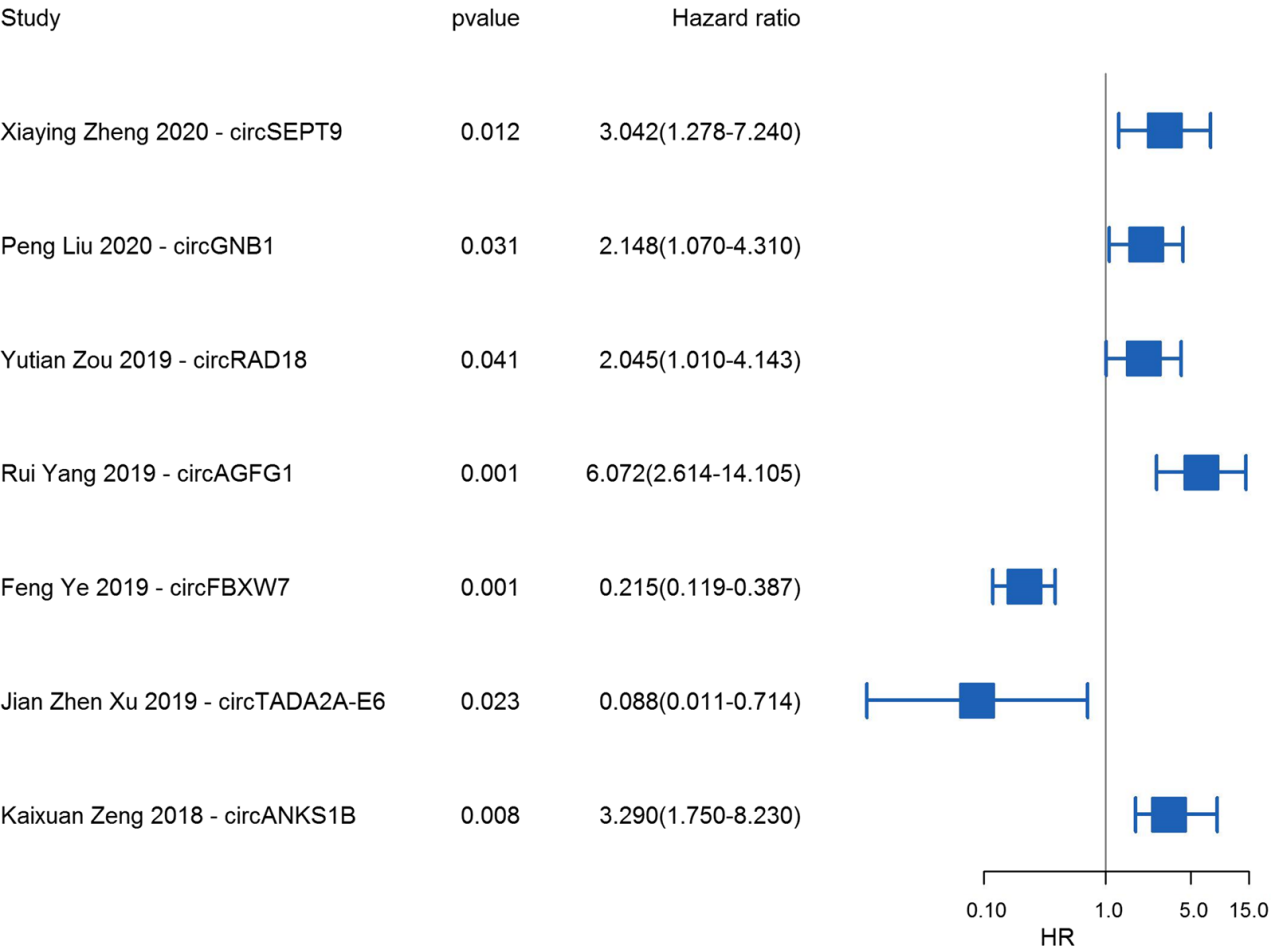
Forest plots of the associations between the expression of circRNAs and TNBC overall survival. Each square indicates a study. *HR* hazard ratio, *CI* confidence interval

21 circRNAs have been reported to have the prognostic values for TNBC patients (Table 4). Specifically, high expression of *circSEPT9*, *circGNB1*, *circRAD18*, *circAGFG1*, and *circANKS1B* and low expression of *circFBXW7* were related to worse survival of TNBC and could be independent prognostic factors for TNBC patients [47, 49, 57, 59, 74, 78], while *circTADA2A-E6* was frequently downreglated in TNBC and whose downregulation were associated with worse survival (Fig. 6). High expression of 15 circRNAs (*circSEPT9*, *circGNB1*, *hsa_circ_0131242*, *circHER2*, *circPGAP3*, *circUBE2D2*, *circRAD18*, *circAGFG1*, *hsa_circ_069718*, *circKIF4A*, *circPLK1*, *circANKS1B*, *circUBAP2*, *circEPSTI1*, and *circGFRA1*) was related to worse OS [46, 47, 49, 51, 53, 54, 57, 59, 63, 64, 75, 78, 79, 81], indicating that they have carcinogenic effects in TNBC. Increased expression of the 5 circRNAs (*circUSP42*, *circFBXW7*, *circTADA2A-E6*, *circAHNAK1*, and *circITCH*) was associated with better OS for TNBC patients [48, 52, 56, 74, 98], suggesting that they serve as tumor suppressors. In terms of DFS, elevated expression of 7 circRNAs (*circGNB1*, *circPGAP3*, *circTFCP2L1*, *circKIF4A*, *circPLK1*, *circEPSTI1*, and *circGFRA1*) showed reduced DFS [29, 46, 51, 54, 55, 59, 64], while high *circUSP42*, *circTADA2A-E6*, *circFBXW7*, and *circAHNAK1* expression predicted better DFS [48, 56, 74, 98], implying that they are related to the recurrence or progression of TNBC.

**Table 4:** Summary of significant associations of circRNAs with TNBC survival

CircRNAs	Roles	TNBC Patients	Expression	Method	Sample type	Cut off	Univariate versus multivariate	Survival	HR (95%CI)	Reference
circSEPT9	Oncogenic	80	Up	ISH	Tissue	6	Multivariate	OS	3.042 (1.278–7.240), *P* = 0.012	[49]
circUSP42	Antitumor	30	Down	RT-qPCR	Tissue	Median	Univariate	OS,DFS	KM	[98]
circGNB1	Oncogenic	222	Up	RT-qPCR	Tissue	NR	Multivariate	OS, DFS	OS, 2.148 (1.070–4.310), *P* = 0.031	[59]
hsa_circ_0131242	Oncogenic	120	Up	RT-qPCR	Tissue	NR	Univariate	OS	KM	[79]
circ-HER2	Oncogenic	59	Up	RT-qPCR	Tissue	> Adjacent	Univariate	OS	KM	[75]
circPGAP3	Oncogenic	86	Up	RT-qPCR	Tissue	Median	Univariate	OS,DFS	KM	[64]
circUBE2D2	Oncogenic	66	Up	RT-qPCR	Tissue	Median	Univariate	OS	KM	[63]
circRAD18	Oncogenic	126	Up	RT-qPCR	Tissue	Median	Multivariate	OS	2.045 (1.010–4.143), *P* = 0.041	[78]
circAGFG1	Oncogenic	80	Up	ISH	Tissue	Median	Multivariate	OS	6.072 (2.614–14.105), *P* < 0.001	[57]
circFBXW7	Antitumor	473	Down	RT-qPCR	Tissue	Mean	Multivariate	OS,DFS	OS, 0.215 (0.119–0.387), *P* = 0.001	[74]
circTADA2A-E6	Antitumor	115	Down	RT-qPCR	Tissue	NR	Univariate	OS,DFS	OS, 0.088 (0.011–0.714), *P* = 0.023; DFS, 0.094 (0.012–0.770), *P* = 0.028	[48]
has_circ_069718	Oncogenic	35	Up	RT-qPCR	Tissue	NR	Univariate	OS	KM	[81]
circAHNAK1	Antitumor	136	Down	RT-qPCR	Tissue	Median	Univariate	OS, DFS	KM	[56]
circITCH	Antitumor	91	Down	RT-qPCR	Tissue	Median	Univariate	OS	KM	[52]
circTFCP2L1	Oncogenic	32	Up	RT-qPCR	Tissue	− 6.58	Univariate	DFS	KM	[55]
circKIF4A	Oncogenic	240	Up	RT-qPCR	Tissue	Mean	Univariate	OS, DFS	KM	[51]
circPLK1	Oncogenic	240	Up	RT-qPCR	Tissue	Mean	Univariate	OS, DFS	KM	[54]
circANKS1B	Oncogenic	165	Up	RT-qPCR	Tissue	Median	Multivariate	OS	3.29 (1.75–8.23), *P* = 0.008	[47]
circUBAP2	Oncogenic	78	Up	RT-qPCR	Tissue	Median	univariate	OS	KM	[53]
circEPSTI1	Oncogenic	240	Up	ISH	Tissue	NR	univariate	OS, DFS	KM	[46]
circGFRA1	Oncogenic	222	Up	RT-qPCR	Tissue	Mean	univariate	OS, DFS	KM	[29]

*NBC* triple-negative breast cancer, *OS* overall survival, *DFS* disease-free survival, *NR* not report, *qRT-PCR* quantitative real-time PCR, *ISH* in situ hybridization, *HR* hazard ratio, *CI* confidence interval

## Conclusions and perspective

TNBC represents a more malignant and aggressive subtype of BC that lacks of effective targeted therapies, and the specific pathogenesis of TNBC is still not fully understood. CircRNAs, previously thought to be the products of RNA splicing errors, are now regarded as an emerging vital player with intriguing functions during various physiological and pathophysiological processes. As discussed in this review, the exact mechanisms of circRNAs maturation have not been fully elucidated, while the dysregulation of circRNAs is believed to be one of the important mechanisms leading to the development and progression of TNBC. As regulators of gene expression, circRNAs are involved in various biological processes of TNBC, including cell proliferation, apoptosis, cell cycle, angiogenesis, metastasis and chemoresistance, making them potential promising biomarkers for TNBC in regarding to diagnosis, prognosis or treatment.

Besides, there are several gaps in the research field of circRNA in TNBC, which need to be further fulfilled in the future. CircRNAs are always maintained at a relatively stable state in cells and they live long in the extracellular environment owing to their unique structures that are resistant to exonuclease RNase Rand [32, 99], identification of dysregulated circRNAs in body fluids, therefore, may be more beneficial for the diagnosis and prognosis of TNBC. Currently, circRNAs in clinical samples, such as plasma, serum, or exosomes have been found to be serve as significant biomarkers in tumor. For example, Wang et al. have identified and validated a number of dysregulated circRNAs in exosomes from BC patients [100] and *circUBE2D2* was found to significantly load in exosomes isolated from tamoxifen-resistant cells, which reinforced tamoxifen resistance in BC [101]. Nevertheless, there is no literature reporting the circulating circRNAs (from blood, urine, saliva, etc.) in TNBC, which should be pursued in future researches. Additionally, the well-known mechanism of circRNAs exerting functions in biological or pathological processes is through ceRNA to target downstream genes, while other potent molecular mechanisms of circRNAs involved in TNBC progression is limited and needs further investigation. Moreover, other unexcavated circRNAs related to TNBC development and progression and elucidation of their corresponding functions are also awaiting for discovery.

Overall, this review gives a systematically summary of the biogenesis, regulatory mechanisms, and biological functions of circRNAs in TNBC, and lists almost all of the circRNAs that dysregulated in TNBC and discusses their significant values for TNBC in regarding to diagnosis, prognosis and chemoresistance, which provides great guiding significance for future researches of circRNAs in TNBC. A better understanding of circRNAs in TNBC may contribute to the development of more reliable diagnosis and treatment straategies for TNBC.

## Supplementary Information

**Additional file 1.** Summary of the relationship between circRNAs expression and the clinicopathological factors of TNBC.

**Additional file 2.** Forest plots of the associations between the expression of circRNAs and **a** age (older vs young), **b** menopause (Yes vs No) of TNBC patients. Each square indicates a study.

## Data Availability

Not applicable.

## Abbreviations

BC: Breast cancer
TNBC: Triple-negative breast cancer
ER: Estrogen receptor
PR: Progesterone receptor
HER2: Human epidermal growth factor receptor 2
TAMs: Tumor-associated macrophages
miRNAs: MicroRNAs
lncRNAs: Long non-coding RNA
circRNAs: Circular RNAs
RNA-seq: RNA sequencing
qRT-PCR: Quantitative real-time PCR
pre-mRNAs: Precursor messenger RNAs
EcircRNAs: Exon circRNAs
ciRNAs: Circular intronic RNAs
EIciRNAs: Exon–intron circRNAs
f-circRNAs: Fusion circRNAs
RBPs: RNA-binding proteins
ceRNAs: Competing endogenous RNAs
mRNAs: Messenger RNAs
IRES: Internal ribosome entry site
EMT: Epithelial-to-mesenchymal transition
DOX: Doxorubicin
MDA-MB-231/DOX: DOX resistant MDA-MB-231
PTX: Paclitaxel
MDA-MB-231/PTX: PTX resistant MDA-MB-231
5-FU: 5-Fluorouracil
OS: Overall survival
DFS: Disease-free survival
