# Supplementary material for: Regulatory mechanisms, functions, and clinical significance of CircRNAs in triple-negative breast cancer
Source: J Hematol Oncol. 2021 Mar 6;14:41. doi: 10.1186/s13045-021-01052-y (PMC7937293; doi:10.1186/s13045-021-01052-y)
Supplement: Supplementary file 1 — Additional file 1. Summary of the relationship between circRNAs expression and the clinicopathological factors of TNBC. [file 13045_2021_1052_MOESM1_ESM.docx]

**Supplementary Table S1. P-values of the correlations between circRNAs expression and TNBC clinicopathological features.**

| **CircRNAs** | **Function** | **Number of patients** | **Expression in tumor** | **Method** | **Sample type** | **Age** | **Menopause** | **Tumor size** | **LN metastasis** | **Histological grade** | **TNM stage** | **T stage** | **Reference** |
| --- | --- | --- | --- | --- | --- | --- | --- | --- | --- | --- | --- | --- | --- |
| circRPPH1 | Oncogenic | 20 | Up | RT-qPCR | tissue | >0.05 | >0.05 | >0.05 | **< 0.05** | NR | >0.05 | NR | (1) |
| circSEPT9 | Oncogenic | 60 | Up | RT-qPCR | tissue | 0.301 | NR | NR | **0.028** | 0.253 | **0.001** | **0.002** | (2) |
| circSEPT9 | Oncogenic | 80 | Up | ISH | tissue | 0.232 | NR | NR | 0.129 | 0.501 | **0.002** | **0.009** | (2) |
| circUSP42 | Antitumor | 30 | Down | RT-qPCR | tissue | 0.464 | 0.731 | NR | **0.005** | 0.456 | **0.032** | NR | (3) |
| circGNB1 | Oncogenic | 222 | Up | RT-qPCR | tissue | 0.563 | 0.766 | **0.001** | 0.072 | NR | **0.007** | NR | (4) |
| hsa_circ_0131242 | Oncogenic | 120 | Up | RT-qPCR | tissue | 0.847 | NR | **<0.001** | NR | NR | **<0.001** | NR | (5) |
| circPGAP3 | Oncogenic | 86 | Up | RT-qPCR | tissue | 0.307 | 0.659 | **0.008** | **0.000** | NR | **0.001** | NR | (6) |
| circUBE2D2 | Oncogenic | 66 | Up | RT-qPCR | tissue | NR | NR | NR | **<0.01** | NR | **<0.01** | NR | (7) |
| circRAD18 | Oncogenic | 126 | Up | RT-qPCR | tissue | 0.471 | 0.278 | **0.002** | 0.109 | 0.069 | **0.010** | **0.001** | (8) |
| hsa_circ_069718 | Oncogenic | 35 | Up | RT-qPCR | tissue | NR | NR | NR | **< 0.05** | NR | **< 0.05** | NR | (9) |
| circFBXW7 | Antitumor | 473 | Down | RT-qPCR | tissue | 0.061 | NR | **0.014** | **0.004** | NR | 0.302 | NR | (10) |
| circAGFG1 | Oncogenic | 40 | Up | RT-qPCR | tissue | 0.342 | 0.204 | NR | **0.025** | 0.197 | 0.212 | **0.027** | (11) |
| circAGFG1 | Oncogenic | 80 | Up | ISH | tissue | 0.816 | NR | NR | 0.260 | **0.004** | 0.329 | 0.152 | (11) |
| circTADA2A-E6 | Antitumor | 115 | Down | RT-qPCR | tissue | 0.548 | NR | NR | **0.012** | NR | **0.022** | 0.697 | (12) |
| circTADA2A-E5/E6 | Antitumor | 115 | Down | RT-qPCR | tissue | 0.819 | NR | NR | 0.623 | NR | 0.660 | 0.551 | (12) |
| circAHNAK1 | Antitumor | 136 | Down | RT-qPCR | tissue | 0.393 | 1.000 | NR | **0.001** | NR | **<0.001** | **0.045** | (13) |
| circITCH | Antitumor | 91 | Down | RT-qPCR | tissue | 0.310 | 0.466 | **0.016** | **0.008** | 0.267 | **0.002** | NR | (14) |
| circTFCP2L1 | Oncogenic | 32 | Up | RT-qPCR | tissue | 0.668 | NR | 0.154 | NR | 0.332 | **0.031** | NR | (15) |
| circKIF4A | Oncogenic | 240 | Up | RT-qPCR | tissue | 0.078 | 0.109 | **<0.001** | **<0.001** | NR | **0.012** | NR | (16) |
| circPLK1 | Oncogenic | 240 | Up | RT-qPCR | tissue | 0.278 | 0.363 | **<0.001** | **<0.001** | NR | **0.010** | NR | (17) |
| circANKS1B | Oncogenic | 165 | Up | RT-qPCR | tissue | 0.454 | 0.576 | 0.180 | **0.004** | 0.158 | **0.013** | NR | (18) |
| circUBAP2 | Oncogenic | 78 | Up | RT-qPCR | tissue | 0.784 | 0.247 | **0.003** | **0.002** | 0.187 | **0.005** | NR | (19) |
| circ-ciRS-7 | Oncogenic | 164 | Up | RT-qPCR | tissue | 0.609 | NR | NR | **0.028** | 0.844 | NR | **0.000** | (20) |
| circEPSTI1 | Oncogenic | 240 | Up | ISH | tissue | 0.285 | 0.375 | **<**0.001 | **0.004** | 0.640 | **0.008** | NR | (21) |
| circGFRA1 | Oncogenic | 222 | Up | RT-qPCR | tissue | 0.216 | 0.738 | **0.029** | **<0.001** | **0.036** | NR | NR | (22) |

TNBC, triple-negative breast cancer; LN, lymph node; qRT-PCR, quantitative real-time PCR; ISH, in situ hybridization; NR, not report.

**Supplementary Table S2. Odds ratio of the correlations between circRNAs expression and TNBC clinicopathological features.**

| **Clinicopathological features** | **CircRNAs** | **High expression** | | **Low expression** | |  |  |  |  |
| --- | --- | --- | --- | --- | --- | --- | --- | --- | --- |
|  |  | **tevent** | **tnoevent** | **cevent** | **cnoevent** | **OR** | **OR. 95L** | **OR.95H** | **P-value** |
| **Age** |  |  |  |  |  |  |  |  |  |
| Yehui Zhou 2020 (≥55 vs < 55) | circRPPH1 | 7 | 4 | 5 | 4 | 1.400 | 0.232 | 8.464 | 0.714 |
| Xiaying Zheng 2020 (≥53 vs < 53) | circSEPT9 | 18 | 12 | 14 | 16 | 1.714 | 0.616 | 4.772 | 0.301 |
| Xiaying Zheng 2020 (≥53 vs < 53) | circSEPT9 | 21 | 22 | 23 | 14 | 0.581 | 0.238 | 1.420 | 0.232 |
| Jinling Yu 2020 (>50 vs ≤ 50) | circUSP42 | 8 | 7 | 6 | 9 | 1.714 | 0.403 | 7.292 | 0.464 |
| Peng Liu 2020 (>50 vs ≤ 50) | circGNB1 | 52 | 32 | 82 | 58 | 1.149 | 0.660 | 2.000 | 0.563 |
| Yueting Li 2020 (≥50 vs < 50) | hsa_circ_0131242 | 59 | 27 | 24 | 10 | 0.910 | 0.383 | 2.167 | 0.847 |
| Dabao He 2020 (>40 vs ≤ 40) | circPGAP3 | 31 | 12 | 35 | 8 | 0.509 | 0.214 | 1.632 | 0.307 |
| Yutian Zou 2019(>50 vs ≤ 50) | circRAD18 | 29 | 34 | 25 | 38 | 1.296 | 0.639 | 2.630 | 0.471 |
| Feng Ye 2019 (>50 vs ≤ 50) | circFBXW7 | 78 | 114 | 140 | 141 | 0.689 | 0.476 | 0.999 | 0.061 |
| Rui Yang 2019(≥50 vs < 50) | circAGFG1 | 11 | 9 | 8 | 12 | 1.833 | 0.522 | 6.434 | 0.342 |
| Rui Yang 2019 (≥50 vs < 50) | circAGFG1 | 25 | 15 | 26 | 14 | 0.897 | 0.360 | 2.234 | 0.816 |
| Weikai Xiao 2019 (≥40 vs < 40) | circAHNAK1 | 48 | 20 | 53 | 15 | 0.679 | 0.313 | 1.474 | 0.393 |
| S. T. WANG 2019 (>40 vs ≤ 40) | circITCH | 31 | 14 | 36 | 10 | 0.615 | 0.240 | 1.579 | 0.310 |
| Qian Wang 2019 (≥55 vs < 50) | circTFCP2L1 | 15 | 9 | 5 | 3 | 1.000 | 0.192 | 5.222 | 0.668 |
| Hailin Tang 2019 (≥50 vs < 50) | circKIF4A | 50 | 50 | 54 | 86 | 1.593 | 0.948 | 2.676 | 0.078 |
| Yanan Kong 2019 (≥50 vs < 50) | circPLK1 | 47 | 52 | 57 | 84 | 1.332 | 0.793 | 2.237 | 0.278 |
| Kaixuan Zeng 2018(>40 vs ≤ 40) | circANKS1B | 68 | 14 | 65 | 18 | 1.345 | 0.619 | 2.925 | 0.454 |
| shengting Wang 2018 (>40 vs ≤ 40) | circUBAP2 | 31 | 8 | 30 | 9 | 1.163 | 0.396 | 3.411 | 0.784 |
| Meixiang Sang 2018 (≥50 vs < 50) | circ-ciRS-7 | 36 | 20 | 65 | 43 | 1.191 | 0.610 | 2.324 | 0.609 |
| Bo Chen 2018 (>50 vs ≤ 50) | circEPSTI1 | 29 | 54 | 66 | 91 | 0.740 | 0.427 | 1.285 | 0.285 |
| Rongfang He 2017 (>50 vs ≤ 50) | circGFRA1 | 44 | 75 | 30 | 73 | 1.428 | 0.811 | 2.512 | 0.216 |
| **Menopause**（Yes vs No） |  |  |  |  |  |  |  |  |  |
| Yehui Zhou 2020 | circRPPH1 | 6 | 5 | 5 | 4 | 0.960 | 0.163 | 5.643 | 0.964 |
| Jinling Yu 2020 | circUSP42 | 9 | 6 | 8 | 7 | 1.313 | 0.309 | 5.583 | 0.731 |
| Peng Liu 2020 | circGNB1 | 51 | 33 | 81 | 57 | 1.088 | 0.625 | 1.892 | 0.766 |
| Dabao He 2020 | circPGAP3 | 16 | 27 | 18 | 25 | 0.823 | 0.346 | 1.956 | 0.659 |
| Yutian Zou 2019 | circRAD18 | 23 | 40 | 29 | 34 | 0.674 | 0.330 | 1.375 | 0.278 |
| Rui Yang 2019 | circAGFG1 | 13 | 7 | 9 | 11 | 2.270 | 0.636 | 8.106 | 0.204 |
| Weikai Xiao 2019 | circAHNAK1 | 27 | 41 | 27 | 41 | 1.000 | 0.503 | 1.988 | 1.000 |
| S. T. WANG 2019 | circITCH | 23 | 22 | 20 | 26 | 1.359 | 0.595 | 3.103 | 0.466 |
| Hailin Tang 2019 | circKIF4A | 46 | 54 | 50 | 90 | 1.533 | 0.908 | 2.588 | 0.109 |
| Yanan Kong 2019 | circPLK1 | 43 | 56 | 53 | 88 | 1.275 | 0.755 | 2.152 | 0.363 |
| Kaixuan Zeng 2018 | circANKS1B | 34 | 48 | 38 | 45 | 0.839 | 0.453 | 1.553 | 0.576 |
| shengting Wang 2018 | circUBAP2 | 13 | 26 | 18 | 21 | 0.583 | 0.233 | 1.458 | 0.247 |
| Bo Chen 2018 | circEPSTI1 | 30 | 53 | 66 | 91 | 0.780 | 0.451 | 1.351 | 0.375 |
| Rongfang He 2017 | circGFRA1 | 50 | 69 | 41 | 62 | 1.096 | 0.641 | 1.874 | 0.738 |
| **Tumor size (>2 vs** ≤ 2) |  |  |  |  |  |  |  |  |  |
| Yehui Zhou 2020 | circRPPH1 | 8 | 3 | 4 | 5 | 3.333 | 0.515 | 21.584 | 0.409 |
| Xiaying Zheng 2020 | circSEPT9 | 24 | 6 | 12 | 18 | 6.000 | 1.890 | 19.043 | **0.002** |
| Xiaying Zheng 2020 | circSEPT9 | 33 | 10 | 18 | 19 | 3.483 | 1.338 | 9.072 | **0.009** |
| Peng Liu 2020 | circGNB1 | 75 | 9 | 88 | 50 | 4.735 | 2.184 | 10.264 | **0.001** |
| Yueting Li 2020 | hsa_circ_0131242 | 77 | 9 | 21 | 13 | 5.296 | 1.993 | 14.074 | **<0.001** |
| Dabao He 2020 | circPGAP3 | 32 | 11 | 20 | 23 | 3.345 | 1.346 | 8.312 | **0.008** |
| Yutian Zou 2019 | circRAD18 | 56 | 7 | 41 | 22 | 4.293 | 1.675 | 11.001 | **0.002** |
| Yutian Zou 2019 (≥5 vs < 5) | circRAD18 | 25 | 38 | 8 | 55 | 4.523 | 1.844 | 11.093 | **0.001** |
| Feng Ye 2019 | circFBXW7 | 131 | 60 | 221 | 60 | 0.593 | 0.390 | 0.900 | **0.014** |
| Rui Yang 2019 | circAGFG1 | 13 | 7 | 6 | 14 | 4.333 | 1.150 | 16.323 | **0.027** |
| Rui Yang 2019 | circAGFG1 | 30 | 10 | 24 | 16 | 2.000 | 0.769 | 5.198 | 0.152 |
| S. T. WANG 2019 | circITCH | 19 | 26 | 31 | 15 | 0.354 | 0.150 | 0.831 | **0.016** |
| Qian Wang 2019 | circTFCP2L1 | 13 | 11 | 2 | 6 | 3.545 | 0.592 | 21.245 | **0.154** |
| Hailin Tang 2019 | circKIF4A | 85 | 15 | 89 | 51 | 3.247 | 1.699 | 6.207 | **<0.001** |
| Yanan Kong 2019 | circPLK1 | 86 | 13 | 88 | 53 | 3.984 | 2.028 | 7.829 | **<0.001** |
| Weikai Xiao 2019 (≥5 vs < 5) | circAHNAK1 | 3 | 65 | 11 | 57 | 0.239 | 0.064 | 0.900 | **0.045** |
| Meixiang Sang 2018 (≥5 vs < 5) | circ-ciRS-7 | 12 | 44 | 7 | 101 | 3.935 | 1.452 | 10.666 | **0.000** |
| Kaixuan Zeng 2018 | circANKS1B | 19 | 63 | 27 | 56 | 0.626 | 0.314 | 1.245 | 0.180 |
| shengting Wang 2018 | circUBAP2 | 16 | 23 | 29 | 10 | 0.240 | 0.092 | 0.627 | **0.003** |
| Bo Chen 2018 | circEPSTI1 | 76 | 7 | 98 | 59 | 6.536 | 2.825 | 15.123 | **<0.001** |
| Rongfang He 2017 | circGFRA1 | 89 | 30 | 63 | 40 | 1.884 | 1.062 | 3.340 | **0.029** |
| **Histological grade (III vs I + II)** |  |  |  |  |  |  |  |  |  |
| Xiaying Zheng 2020 | circSEPT9 | 10 | 12 | 7 | 17 | 2.024 | 0.600 | 6.829 | 0.253 |
| Xiaying Zheng 2020 | circSEPT9 | 23 | 20 | 17 | 20 | 1.353 | 0.560 | 3.267 | 0.501 |
| Jinling Yu 2020 (III vs II) | circUSP42 | 10 | 5 | 8 | 7 | 1.750 | 0.400 | 7.664 | 0.456 |
| Yutian Zou 2019 | circRAD18 | 30 | 33 | 20 | 43 | 1.955 | 0.946 | 4.036 | 0.069 |
| Rui Yang 2019 (III vs II) | circAGFG1 | 10 | 10 | 6 | 14 | 2.333 | 0.638 | 8.538 | 0.197 |
| Rui Yang 2019 (III vs II) | circAGFG1 | 26 | 14 | 13 | 27 | 3.857 | 1.526 | 9.750 | **0.004** |
| S. T. WANG 2019 | circITCH | 10 | 35 | 16 | 30 | 0.536 | 0.212 | 1.356 | 0.267 |
| Qian Wang 2019 (III+ II vs I) | circTFCP2L1 | 8 | 16 | 4 | 4 | 0.500 | 0.098 | 2.540 | 0.332 |
| Kaixuan Zeng 2018 | circANKS1B | 24 | 58 | 14 | 69 | 2.039 | 0.967 | 4.300 | 0.158 |
| shengting Wang 2018 | circUBAP2 | 19 | 20 | 5 | 34 | 6.460 | 2.088 | 19.986 | 0.187 |
| Meixiang Sang 2018 | circ-ciRS-7 | 19 | 37 | 35 | 73 | 1.071 | 0.540 | 2.123 | 0.844 |
| Bo Chen 2018 | circEPSTI1 | 35 | 48 | 61 | 96 | 1.148 | 0.668 | 1.971 | 0.640 |
| Rongfang He 2017 | circGFRA1 | 67 | 52 | 44 | 59 | 1.728 | 1.014 | 2.943 | **0.036** |
| **LN metastasis (positive vs negative)** |  |  |  |  |  |  |  |  |  |
| Yehui Zhou 2020 | circRPPH1 | 8 | 3 | 2 | 7 | 9.333 | 1.193 | 72.991 | **0.025** |
| Xiaying Zheng 2020 | circSEPT9 | 14 | 16 | 6 | 24 | 3.500 | 1.112 | 11.017 | **0.028** |
| Jinling Yu 2020 | circUSP42 | 1 | 14 | 8 | 7 | 0.063 | 0.006 | 0.604 | **0.005** |
| Peng Liu 2020 | circGNB1 | 50 | 34 | 65 | 73 | 1.652 | 0.954 | 2.860 | 0.072 |
| Dabao He 2020 | circPGAP3 | 36 | 7 | 19 | 24 | 6.496 | 2.369 | 17.815 | **0.000** |
| Yutian Zou 2019 | circRAD18 | 37 | 26 | 28 | 35 | 1.779 | 0.878 | 3.604 | 0.109 |
| Feng Ye 2019 | circFBXW7 | 90 | 101 | 168 | 109 | 0.578 | 0.398 | 0.839 | **0.004** |
| Rui Yang 2019 | circAGFG1 | 15 | 5 | 8 | 12 | 4.500 | 1.166 | 17.373 | **0.025** |
| Rui Yang 2019 | circAGFG1 | 20 | 20 | 15 | 25 | 1.667 | 0.684 | 4.063 | 0.260 |
| Weikai Xiao 2019 | circAHNAK1 | 20 | 48 | 41 | 27 | 0.274 | 0.135 | 0.560 | **0.001** |
| S. T. WANG 2019 | circITCH | 20 | 25 | 33 | 13 | 0.315 | 0.132 | 0.753 | **0.008** |
| Hailin Tang 2019 | circKIF4A | 63 | 37 | 54 | 86 | 2.712 | 1.597 | 4.606 | **<0.001** |
| Yanan Kong 2019 | circPLK1 | 62 | 37 | 55 | 86 | 2.620 | 1.543 | 4.449 | **<0.001** |
| Kaixuan Zeng 2018 | circANKS1B | 46 | 36 | 28 | 55 | 2.510 | 1.336 | 4.714 | **0.004** |
| shengting Wang 2018 | circUBAP2 | 18 | 21 | 31 | 8 | 0.221 | 0.081 | 0.601 | **0.002** |
| Meixiang Sang 2018 | circ-ciRS-7 | 40 | 16 | 58 | 50 | 2.155 | 1.079 | 4.306 | **0.028** |
| Bo Chen 2018 | circEPSTI1 | 51 | 32 | 66 | 91 | 2.197 | 1.276 | 3.785 | **0.004** |
| Rongfang He 2017 | circGFRA1 | 89 | 30 | 16 | 87 | 16.131 | 8.214 | 31.678 | **<0.001** |
| **TNM stage ( III + IV vs I + II)** |  |  |  |  |  |  |  |  |  |
| Yehui Zhou 2020 | circRPPH1 | 6 | 4 | 2 | 8 | 6.000 | 0.812 | 44.351 | 0.068 |
| Xiaying Zheng 2020 ( III + II vs I) | circSEPT9 | 26 | 4 | 14 | 16 | 7.429 | 2.078 | 26.553 | **0.001** |
| Xiaying Zheng 2020 ( III+ II vs I) | circSEPT9 | 39 | 4 | 23 | 14 | 5.935 | 1.744 | 20.200 | **0.002** |
| Jinling Yu 2020 | circUSP42 | 0 | 15 | 4 | 11 | 1.364 | 1.005 | 1.850 | **0.032** |
| Peng Liu 2020 | circGNB1 | 27 | 57 | 23 | 115 | 2.368 | 1.248 | 4.493 | **0.007** |
| Yueting Li 2020 (III+ II vs I) | hsa_circ_0131242 | 76 | 10 | 19 | 15 | 6.000 | 2.332 | 15.436 | **<0.001** |
| Dabao He 2020 ( III vs I + II) | circPGAP3 | 18 | 25 | 5 | 38 | 5.472 | 1.800 | 16.637 | **0.002** |
| Yutian Zou 2019 | circRAD18 | 24 | 39 | 11 | 52 | 2.909 | 1.274 | 6.642 | **0.010** |
| Feng Ye 2019 | circFBXW7 | 67 | 124 | 111 | 168 | 0.818 | 0.558 | 1.198 | 0.302 |
| Rui Yang 2019 ( III vs I + II) | circAGFG1 | 5 | 15 | 2 | 18 | 3.000 | 0.507 | 17.740 | 0.212 |
| Rui Yang 2019 ( III vs I + II) | circAGFG1 | 14 | 26 | 10 | 30 | 1.615 | 0.614 | 4.247 | 0.329 |
| Weikai Xiao 2019 | circAHNAK1 | 4 | 64 | 25 | 43 | 0.108 | 0.035 | 0.331 | **<0.001** |
| S. T. WANG 2019 (III vs I + II) | circITCH | 5 | 40 | 20 | 26 | 0.163 | 0.054 | 0.487 | **0.002** |
| Qian Wang 2019 (III vs I+ II) | circTFCP2L1 | 17 | 7 | 2 | 6 | 7.286 | 1.173 | 45.255 | **0.031** |
| Hailin Tang 2019 | circKIF4A | 30 | 70 | 23 | 117 | 2.180 | 1.174 | 4.047 | **0.012** |
| Yanan Kong 2019 | circPLK1 | 30 | 69 | 23 | 118 | 2.231 | 1.201 | 4.143 | **0.010** |
| Kaixuan Zeng 2018 | circANKS1B | 36 | 46 | 18 | 65 | 2.826 | 1.431 | 5.580 | **0.013** |
| shengting Wang 2018 (III vs I + II) | circUBAP2 | 18 | 21 | 6 | 33 | 4.714 | 1.611 | 13.796 | **0.005** |
| Bo Chen 2018 | circEPSTI1 | 26 | 57 | 26 | 131 | 2.298 | 1.229 | 4.299 | **0.008** |
| Rongfang He 2017 | circGFRA1 | 57 | 62 | 10 | 93 | 8.550 | 4.060 | 18.007 | **<0.001** |

TNBC, triple-negative breast cancer; LN, lymph node.

**Reference**

1. Zhou Y, Liu X, Lan J, Wan Y, Zhu X. Circular RNA circRPPH1 promotes triple-negative breast cancer progression via the miR-556-5p/YAP1 axis. American journal of translational research. 2020;12(10):6220-34.

2. Zheng X, Huang M, Xing L, Yang R, Wang X, Jiang R, et al. The circRNA circSEPT9 mediated by E2F1 and EIF4A3 facilitates the carcinogenesis and development of triple-negative breast cancer. Molecular cancer. 2020;19(1):73.

3. Yu J, Shen W, Xu J, Gong B, Gao B, Zhu J. circUSP42 Is Downregulated in Triple-Negative Breast Cancer and Associated With Poor Prognosis. Technology in cancer research & treatment. 2020;19:1533033820950827.

4. Liu P, Zou Y, Li X, Yang A, Ye F, Zhang J, et al. circGNB1 Facilitates Triple-Negative Breast Cancer Progression by Regulating miR-141-5p-IGF1R Axis. Frontiers in genetics. 2020;11:193.

5. Li Y, Shi P, Zheng T, Ying Z, Jiang D. Circular RNA hsa_circ_0131242 Promotes Triple-Negative Breast Cancer Progression by Sponging hsa-miR-2682. OncoTargets and therapy. 2020;13:4791-8.

6. He D, Yang X, Kuang W, Huang G, Liu X, Zhang Y. The Novel Circular RNA Circ-PGAP3 Promotes the Proliferation and Invasion of Triple Negative Breast Cancer by Regulating the miR-330-3p/Myc Axis. OncoTargets and therapy. 2020;13:10149-59.

7. Dou D, Ren X, Han M, Xu X, Ge X, Gu Y, et al. CircUBE2D2 (hsa_circ_0005728) promotes cell proliferation, metastasis and chemoresistance in triple-negative breast cancer by regulating miR-512-3p/CDCA3 axis. Cancer cell international. 2020;20:454.

8. Zou Y, Zheng S, Xiao W, Xie X, Yang A, Gao G, et al. circRAD18 sponges miR-208a/3164 to promote triple-negative breast cancer progression through regulating IGF1 and FGF2 expression. Carcinogenesis. 2019;40(12):1469-79.

9. Zhang J, Xu HD, Xing XJ, Liang ZT, Xia ZH, Zhao Y. CircRNA_069718 promotes cell proliferation and invasion in triple-negative breast cancer by activating Wnt/beta-catenin pathway. European review for medical and pharmacological sciences. 2019;23(12):5315-22.

10. Ye F, Gao G, Zou Y, Zheng S, Zhang L, Ou X, et al. circFBXW7 Inhibits Malignant Progression by Sponging miR-197-3p and Encoding a 185-aa Protein in Triple-Negative Breast Cancer. Molecular therapy Nucleic acids. 2019;18:88-98.

11. Yang R, Xing L, Zheng X, Sun Y, Wang X, Chen J. The circRNA circAGFG1 acts as a sponge of miR-195-5p to promote triple-negative breast cancer progression through regulating CCNE1 expression. Molecular cancer. 2019;18(1):4.

12. Xu J-Z, Shao C-C, Wang X-J, Zhao X, Chen J-Q, Ouyang Y-X, et al. circTADA2As suppress breast cancer progression and metastasis via targeting miR-203a-3p/SOCS3 axis. Cell death & disease. 2019;10(3):175.

13. Xiao W, Zheng S, Zou Y, Yang A, Xie X, Tang H, et al. CircAHNAK1 inhibits proliferation and metastasis of triple-negative breast cancer by modulating miR-421 and RASA1. Aging. 2019;11(24):12043-56.

14. Wang ST, Liu LB, Li XM, Wang YF, Xie PJ, Li Q, et al. Circ-ITCH regulates triple-negative breast cancer progression through the wnt/β-catenin pathway. Neoplasma. 2019;66(2):232-9.

15. Wang Q, Li Z, Hu Y, Zheng W, Tang W, Zhai C, et al. Circ-TFCP2L1 Promotes the Proliferation and Migration of Triple Negative Breast Cancer through Sponging miR-7 by Inhibiting PAK1. Journal of mammary gland biology and neoplasia. 2019;24(4):323-31.

16. Tang H, Huang X, Wang J, Yang L, Kong Y, Gao G, et al. circKIF4A acts as a prognostic factor and mediator to regulate the progression of triple-negative breast cancer. Molecular cancer. 2019;18(1):23.

17. Kong Y, Yang L, Wei W, Lyu N, Zou Y, Gao G, et al. CircPLK1 sponges miR-296-5p to facilitate triple-negative breast cancer progression. Epigenomics. 2019;11(10):1163-76.

18. Zeng K, He B, Yang BB, Xu T, Chen X, Xu M, et al. The pro-metastasis effect of circANKS1B in breast cancer. Molecular cancer. 2018;17(1):160.

19. Wang S, Li Q, Wang Y, Li X, Wang R, Kang Y, et al. Upregulation of circ-UBAP2 predicts poor prognosis and promotes triple-negative breast cancer progression through the miR-661/MTA1 pathway. Biochemical and biophysical research communications. 2018;505(4):996-1002.

20. Sang M, Meng L, Liu S, Ding P, Chang S, Ju Y, et al. Circular RNA ciRS-7 Maintains Metastatic Phenotypes as a ceRNA of miR-1299 to Target MMPs. Molecular cancer research : MCR. 2018;16(11):1665-75.

21. Chen B, Wei W, Huang X, Xie X, Kong Y, Dai D, et al. circEPSTI1 as a Prognostic Marker and Mediator of Triple-Negative Breast Cancer Progression. Theranostics. 2018;8(14):4003-15.

22. He R, Liu P, Xie X, Zhou Y, Liao Q, Xiong W, et al. circGFRA1 and GFRA1 act as ceRNAs in triple negative breast cancer by regulating miR-34a. Journal of experimental & clinical cancer research : CR. 2017;36(1):145.
