# Supplementary figures and images for: Regulatory mechanisms, functions, and clinical significance of CircRNAs in triple-negative breast cancer
Source: J Hematol Oncol. 2021 Mar 6;14:41. doi: 10.1186/s13045-021-01052-y (PMC7937293; doi:10.1186/s13045-021-01052-y)

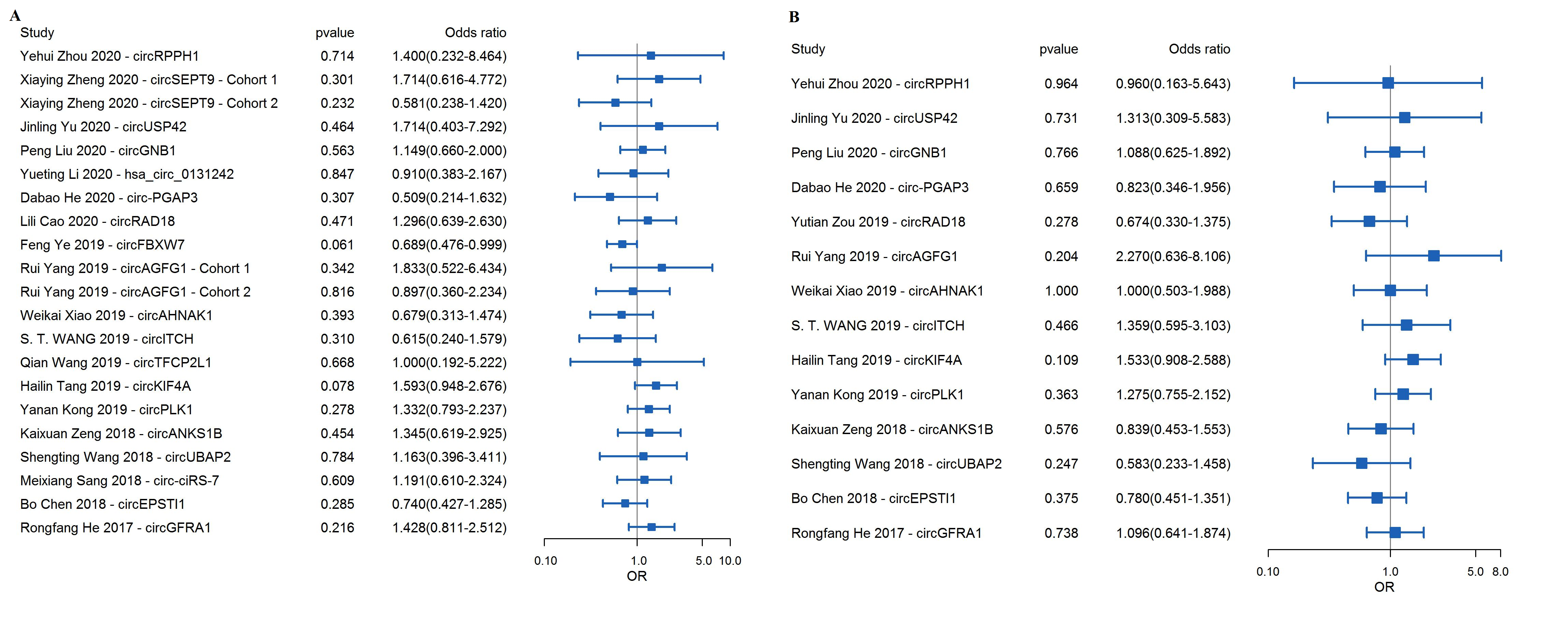

Supplement: Supplementary file 2 — Additional file 2. Forest plots of the associations between the expression of circRNAs and a age (older vs young), b menopause (Yes vs No) of TNBC patients. Each square indicates a study. [file 13045_2021_1052_MOESM2_ESM.tif]
